# Binding of the TRF2 iDDR motif to RAD50 highlights a convergent evolutionary strategy to inactivate MRN at telomeres

**DOI:** 10.1093/nar/gkae509

**Published:** 2024-06-17

**Authors:** Freddy Khayat, Majedh Alshmery, Mohinder Pal, Antony W Oliver, Alessandro Bianchi

**Affiliations:** Genome Damage and Stability Centre, School of Life Sciences, University of Sussex, Brighton, UK; Genome Damage and Stability Centre, School of Life Sciences, University of Sussex, Brighton, UK; Department of Life Sciences, Hafr Al Batin University, Saudi Arabia; Genome Damage and Stability Centre, School of Life Sciences, University of Sussex, Brighton, UK; School of Biosciences, University of Kent, Canterbury, UK; Genome Damage and Stability Centre, School of Life Sciences, University of Sussex, Brighton, UK; Genome Damage and Stability Centre, School of Life Sciences, University of Sussex, Brighton, UK

## Abstract

Telomeres protect chromosome ends from unscheduled DNA repair, including from the MRN (MRE11, RAD50, NBS1) complex, which processes double-stranded DNA breaks (DSBs) via activation of the ATM kinase, promotes DNA end-tethering aiding the non-homologous end-joining (NHEJ) pathway, and initiates DSB resection through the MRE11 nuclease. A protein motif (MIN, for MRN inhibitor) inhibits MRN at budding yeast telomeres by binding to RAD50 and evolved at least twice, in unrelated telomeric proteins Rif2 and Taz1. We identify the iDDR motif of human shelterin protein TRF2 as a third example of convergent evolution for this telomeric mechanism for binding MRN, despite the iDDR lacking sequence homology to the MIN motif. CtIP is required for activation of MRE11 nuclease action, and we provide evidence for binding of a short C-terminal region of CtIP to a RAD50 interface that partly overlaps with the iDDR binding site, indicating that the interaction is mutually exclusive. In addition, we show that the iDDR impairs the DNA binding activity of RAD50. These results highlight direct inhibition of MRN action as a crucial role of telomeric proteins across organisms and point to multiple mechanisms enforced by the iDDR to disable the many activities of the MRN complex.

## Graphical Abstract

**Graphical Abstract:**
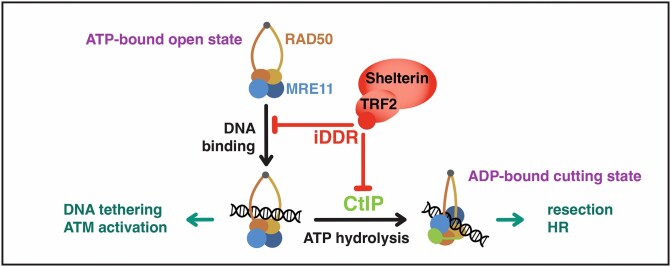

## Introduction

Eukaryotes have evolved specialized structures, the telomeres, to protect the ends of their segmented linear genomes. Protection requires maintenance and replication of the telomeres themselves, by the telomerase enzyme, and concomitant inhibition of DNA repair pathways, such as DNA double-strand break (DSB) repair, that would otherwise compromise integrity of the ends (1,2). To execute these functions telomeres possess a number of conserved features, which include tandem arrays of DNA repeats, mostly configured as double-stranded DNA but with a terminal single-stranded 3′ overhang. These repeats are bound by a set of DNA double- and single-stranded telomeric DNA binding factors that assemble a protein complex at the chromosome ends (3,4). Despite these general similarities, the exact protein make-up of this complex is highly variable within eukaryotes, suggesting a scenario where gene duplications and divergence within the telomeric repeats—due to mutations in the telomerase RNA template—have led to diversity in the set, arrangement, and number of telomere associated factors (5–9). This points to a highly malleable complex where the main feature is the presence of a conserved set of functionalities but within a great degree of architectural variation and structural flexibility, a notion that has been confirmed experimentally (10).

The core set of telomeric factors in metazoans is constituted by shelterin, which in humans is made of six protein factors (4), whereas in budding yeast the telomeric complex takes a markedly different form (11). In all cases, however, the action of additional factors—whose function is not primarily telomeric—is co-opted at telomeres. A case in point is represented by the MRN complex itself and the two main kinases involved in the DNA damage response (DDR), ATM and ATR. MRN (MRX in budding yeast, comprising of RAD50, MRE11 and NBS1/Xrs2) acts as the main sensor of DSBs (12,13) and is responsible for their resection via the endonucleolytic action of the MRE11 nuclease, which requires also the ATPase activity of RAD50, and the CtIP/Sae2/Ctp1 co-factor (14–16). In addition, MRN is required for activation of the ATM/Tel1 kinase (17–19). In yeast, the MRX complex is additionally required for DSB repair by NHEJ (20,21), an activity that has been attributed to the ability of the long coiled-coil stemming from the globular head domain of RAD50 to interact intermolecularly and to promote DNA end-tethering (22,23). Although telomeres suppress the ability of MRN to promote DNA repair, in budding yeast MRX/Tel1 also plays an important role in activating telomerase (24) whilst in human cells ATM can similarly promote telomerase action (25,26). Elegant experiments carried out in yeast have shown that MRX/Tel1 is responsible for resection and telomerase recruitment at telomeres replicated by the leading strand, likely due to the presence of a blunt end after DNA replication (27,28). Intriguingly, at mammalian telomeres leading telomere resection and overhang-establishment are not executed by MRN but by the Apollo nuclease (29). Telomeres therefore participate in a delicate and balancing act serving to regulate MRN activity.

The telomeric protein complex in the budding yeast *Saccharomyces cerevisiae* is assembled from the DNA binding protein Rap1 which, among other things, recruits the telomere factor Rif2, which originated from duplication of the essential Orc4 gene in a subset of yeast clades (30). Rif2 was shown to be part of a Rap1 pathway leading to inhibition of Tel1 in promoting telomere length (31), with work from the Longhese laboratory providing an early glimpse of the ability of Rif2 to counteract Tel1, by showing that overexpression of Rif2 is sufficient to suppress hyperactivation of Tel1 (32). Further work has demonstrated that Rif2 acts to ‘mark’ short telomeres for preferential elongation by telomerase (33), likely by counteracting increased activation of Tel1 at shortened telomeres (34–36).

More recent work has shed light on a possible mechanism for the control of MRN activity at yeast telomeres by Rif2, through identification of a short amino acid motif within the protein (called MIN, for MRN-inhibitor, or BAT, for blocks the addition of telomeres) that is capable of disabling MRN activity in DNA resection, in DNA repair by NHEJ, and in the activation of Tel1 (37–40). The MIN motif acts by binding to the RAD50 subunit, to an exposed β-sheet that is also responsible for making contact with Sae2 (37,38,41). Within the MRN complex, RAD50 fulfils a role as a crucial regulatory subunit, responsible for orchestrating an ATP-driven shift from a ‘resting’ ATP-bound conformation to a ‘cutting’ one that is ADP-bound (42,43). While the former is proficient for DNA binding and tethering, as well as for Tel1/ATM activation and for NHEJ, the latter confers nuclease activity (22,44). The MIN motif could in principle disable nucleolytic action by displacing Sae2 from MRN, and counter NHEJ and Tel1 activation by interfering with the ‘resting’ conformation, possibly in light of its ability to increase the rate of ATP hydrolysis by RAD50 (37,38,40,41,45).

Although the importance of MRN in processing telomeric ends under physiological conditions might vary in different organisms, it is clear that MRN has a unique role in activating ATM at deprotected telomeres, for example after loss of shelterin subunit TRF2 (46–48). TRF2 plays a key role in orchestrating multiple aspects of mammalian telomere protection, associating with the MRN complex via a highly conserved amino acid motif (called iDDR, for inhibitor of DDR) which is located in an unstructured region nestled between the TRF2 homodimerization (TRFH) and DNA binding motifs, and which suppresses the DDR at deprotected telomeres (49). Because TRF2 interacts directly with NBS1 through its TRFH domain, in a manner regulated by NBS1 phosphorylation (50), and also with ATM (51), it has been unclear whether TRF2, or any other shelterin subunit, might act to regulate MRN at telomeres using a strategy similar to that employed by the MIN motif of yeast Rif2. Recent work has identified a role for the iDDR motif of TRF2 in controlling MRN-dependent resection at leading-end telomeres, in concert with DNA-PK, and in inhibiting the endonuclease action of MRN *in vitro* (52), proposing like us that these functions are carried out via binding to RAD50 (53). Here we address this question, showing that the iDDR motif of TRF2 binds to RAD50 in a manner similar to that of the MIN motif, and provide a comprehensive analysis and validation of the interaction model. In addition, we define and characterize the interaction of CtIP with RAD50 and show that it has requirements that are incompatible with iDDR-bound RAD50. Finally, we show that the MRN-inhibiting capabilities of the iDDR extend to include its ability to interfere with DNA binding by RAD50, underscoring another point of similarity with the yeast MIN motif, which interferes with multiple aspects of MRN action (17,37,38,41,54). The binding of the iDDR of TRF2 to RAD50 represents the third example that we have documented for the convergent evolution of the interaction of telomeric proteins with a specific region of RAD50, with the ability to inhibit the action of MRN at telomeres. The characterization of this recurring mechanism identifies what appears to be an ancestral role for telomeric proteins and sheds new light on the regulatory opportunities of the multi-purpose MRN complex.

## Materials and methods

### Plasmids

Plasmids for yeast two-hybrid **(**Y2H) analysis were constructed in pGBKT7 or pGADT7, or in modified vectors for either N- or C-terminal tagging. Mutants were created by PCR with the desired changes embedded in the PCR primers, followed by incorporation of one or two fragments into the appropriate vectors by Gibson Assembly using home-made reaction mixes, or by cloning of synthetic fragments (Integrated DNA Technologies), also by Gibson Assembly (55). All clones were verified by sequencing.

### Yeast two-hybrid analysis

Strains were grown in synthetic complete (SC) minimal medium (2% w/v glucose, 0.67% w/v yeast nitrogen base, 0.2% w/v SC dropout mix, 2% w/v agar, pH 6.0). YNB and SC mixes were from United States Biological. For transformations, the LiAc protocol was followed, with heat shock at 42°C for 45 min. Yeast two hybrid assays were performed by transforming GBD (Gal4 DNA Binding Domain) and GAD (Gal4 Activating Domain) plasmids in the haploid budding yeast strains Y2HGold (YAB2356) and Y187 (YAB2357), respectively. YAB2356: MATa trp1-901 leu2-3112 ura3-52 his3-200 gal4Δ gal80Δ LYS2 : : GAL1_UAS_–Gal1_TATA_–His3 GAL2_UAS_–Gal2_TATA_–Ade2 URA3::MEL1_UAS_–Mel1_TATA_ AUR1-C MEL1. YB2357: MATα ura3-52 his3-200 ade2-101 trp1-901 leu2-3112 gal4Δ gal80Δ met–URA3::GAL1_UAS_–Gal1_TATA_–LacZ MEL1. GBD and GAD transformants were selected on SC medium lacking tryptophan or leucine, respectively. Haploid strains were then mated on rich-medium YPAD plates, and diploids were selected after one day by streaking on plates lacking both tryptophan and leucine. Interactions were assessed by quantification of expression of the HIS3 and ADE2 markers by spotting 5-fold dilutions of liquid cultures on minimal medium lacking, in addition, histidine or adenine, starting with 20000 cells. Plates were incubated at 30°C for 2–3 (SC-leu-trp) or 3–4 (selective plates) days.

### AlphaFold2 models

All models were generated using ColabFold v1.5.2: AlphaFold2 using MMseqs2 (56). In each case, five amber-relaxed models were generated, with no specified or provided template. The option to use the ‘alphafold2_multimer_v3’ model type was selected. All other parameters were left at their default settings. To reduce computational ‘cost’ the amino acid sequence corresponding to just the head-domain of RAD50 was provided in each case: consisting of the N-terminal portion and a short (∼44 amino acid) section of the ascending helix ‘fused’ to a similar section of the descending helix and C-terminal portion by an artificial linking segment consisting of the amino acid sequence ‘GGGGGGSGGGGGGSGGGGGG’.

### Molecular graphics

Figures were generated using either PyMOL (v. 2.5.4) (57) (https://pymol.org/2) or ChimeraX (v. 1.1.1) (58). PyMOL was used to colour models according to the predicted local distance difference test (pLLDT) scores present in the *B*-factor column of the PDB file generated for each model, using a ‘reverse rainbow spectrum’ ranging from blue (pLDDT score > 80, high confidence) to red (pLDDT score < 50, low confidence or intrinsically disordered region). Electrostatic surfaces were calculated using the inbuilt functionality of ChimeraX.

### Expression constructs

All synthetic gene fragments were codon-optimised for expression in *Escherichia coli* and purchased from GeneArt (ThermoFisher Scientific, Waltham, USA). Expression constructs were verified by nanopore sequencing (Full Circle Labs, Imperial College, London). For expression of HsRad50-head two synthetic gene fragments encoding amino acids (aa) 1113–1312 and 1–200 of human RAD50 (UniProt ID: RAD50_HUMAN) were cloned into pRSF-1b between restriction sites NcoI and XmaJI using Gibson assembly. To generate a polycistronic expression system both fragments contained an in-frame upstream ribosome binding site. In addition, a human rhinovirus 3C-cleavage site (3C) followed by 2 × STREP-II affinity tag was placed at the C-terminus of the aa 1–200 fragment, followed by a T7 terminator sequence. For expression of GST-iDDR(WT) and GST-iDDR(W465A) synthetic gene fragments encoding a Myc epitope tag, an extended linker sequence, and TRF2 aa 449–473 (UniProt ID: TERF2_HUMAN) were cloned into the NdeI and XhoI restriction sites of pTHREE-E using Gibson assembly. pTHREE-E is an in-house modified version of pET-17b which encodes an N-terminal, 3C-cleavable fusion with GST. For expression of TRX-iDDR(WT) and TRX-CtIP(WT) synthetic gene fragments encoding aa 449–473 of human TRF2 and aa 844–890 of human CtIP (UniProt ID: CTIP_HUMAN) were cloned into the ApaI and EcoRI restriction sites of pAWO-His-TRX using Gibson assembly. pAWO-His-TRX is an in-house modified version of pET-17b which encodes an N-terminal His-tagged, 3C-cleavable, fusion with E. coli Thioredoxin (TrxA).

### Protein expression and purification

Buffers: A, 50 mM Bis–Tris Propane pH 8.0, 300 mM NaCl, 0.5 mM TCEP, 5% v/v glycerol; B, 50 mM Bis–Tris propane pH 8.0, 1 M NaCl, 0.5 mM TCEP, 5% v/v glycerol; C, 50 mM Bis–Tris propane pH 8.0, 300 mM NaCl, 0.5 mM TCEP, 5% v/v glycerol 2.5 mM desthiobiotin; D, 25 mM Bis–Tris propane pH 8.0, 300 mM NaCl, 0.5 mM TCEP, 5% v/v glycerol; E, 50 mM HEPES·NaOH pH 7.5, 250 mM NaCl, 10 mM imidazole, 0.5 mM TCEP; F, 50 mM HEPES·NaOH pH 7.5, 250 mM NaCl, 300 mM imidazole, 0.5 mM TCEP; G, 25 mM Bis–Tris propane pH 8.0, 200 mM NaCl, 0.5 mM TCEP, 5% v/v glycerol; H, 50 mM HEPES·NaOH pH 7.5, 250 mM NaCl, 0.5 mM TCEP; I, 50 mM HEPES·NaOH pH 7.5, 1 M NaCl, 0.5 mM TCEP; J, 50 mM HEPES·NaOH pH 7.5, 250 mM NaCl, 0.5 mM TCEP, 20 mM glutathione; K, 25 mM HEPES·NaOH pH 7.5 200 mM NaCl 0.5 mM TCEP.

HsRad50-head. The cell pellet from 1 litre of cell culture was resuspended on ice, in BUFFER A supplemented with protease inhibitors (Roche, Burgess Hill, UK). Cells were lysed though a combination of the thawing process and sonication, and insoluble material removed by high-speed centrifugation at 40 000 × g for a period of 1 h at 4°C. The soluble supernatant was then filtered through a 5 μm filter (Sartorius Stedim, Epsom, UK) then applied to a 5 ml HiTrap Strep column (Cytiva) pre-equilibrated in BUFFER A, at a flow rate of 1 ml/min. The column was washed with 5 column volumes (CV) of BUFFER A, followed by 5 CV wash with BUFFER B then 5 CV of BUFFER A, the retained protein eluted by application of 5 CV of BUFFER C in 1 CV fractions. The most concentrated fraction was then loaded on a Superdex 75 26 600 size exclusion chromatography column pre-equilibrated with BUFFER D. Pooled fractions were concentrated to ∼125 μM using a 5000 MWCO concentrator (Sartorius Stedim, Epsom, UK) and stored at –80°C until required.

GST-iDDR(WT) and GST-iDDR(W465A). The cell pellet from 1 l of cell culture was resuspended on ice, in BUFFER H supplemented with protease inhibitors (Roche, Burgess Hill, UK). Cells were lysed though a combination of the thawing process and sonication, and insoluble material removed by high-speed centrifugation at 40 000 × g for a period of 1 h at 4°C. The soluble supernatant was then filtered through a 5 μm filter then applied to a batch/gravity column containing 5 ml Amintra Glutathione resin (Expedeon, Over, UK), pre-equilibrated in BUFFER H. The resin was washed with 5 column volumes (CV) of BUFFER H, followed by 5 CV wash with BUFFER I then 5 CV of BUFFER H, the retained protein eluted by application of 5 CV of BUFFER J in 1 CV fractions. The most concentrated fractions were pooled, then loaded on a Superdex 200 26 600 size exclusion chromatography column pre-equilibrated with BUFFER K. Pooled fractions were concentrated using a 5000 MWCO concentrator and stored at –80°C until required. Throughout the purification procedure, samples were analysed by SDS-PAGE in order to monitor yield and purity.

TrxA-iDDR(WT) and TRX-CtiP(WT). The cell pellet from 1 l of cell culture was resuspended on ice, in BUFFER E supplemented with protease inhibitors. Cells were lysed though a combination of the thawing process and sonication, and insoluble material removed by high-speed centrifugation at 40 000 × g for a period of 1 hour at 4°C. The soluble supernatant was then filtered through a 5 μm filter (then applied to a batch/gravity column containing 2 ml TALON Metal Affinity Resin (Takara), pre-equilibrated in BUFFER E. The resin was washed with 10 column volumes (CV) of BUFFER E. The retained protein eluted by application of 5 CV of BUFFER F in 1 CV fractions. The most concentrated fractions were pooled, then loaded on a Superdex 75 26 600 size exclusion chromatography column pre-equilibrated with BUFFER G. Pooled fractions were concentrated using a 5000 MWCO concentrator and stored at –80°C until required.

### Electrophoretic mobility shift assays

DNA duplexes were generated by annealing an equimolar amount of purified oligonucleotides (5′-F-TAGTGCTGTAGGAGAATATACGGGCTGCTCGTGTTGACAAGTACTGAT-3′ and 5′-ATCAGTACTTGTCAACACGAGCAGCCCTATATTCTCCTACAGCACTA-3′where F = 5-carboxyfluorescein, purchased from Merck, Darmstadt, Germany), to yield a final concentration of 100 MM. All EMSA experiments were carried out in 20 mM Bis–Tris propane pH 8.0, 200 mM NaCI, 0.5 mM TCEP, with each sample containing 500 nM of annealed DNA duplex. Mg-ATP was added to a 50 mM solution of HsRAD50-head (250 μM final concentration) just prior to use. DNA and other recombinant proteins were then added. Sample volumes were typically 12 μl, with 4.5 μl representing protein component ‘A’, 4.5 μl component B, 0.5 μl DNA and 2 μl of 40% w/v sucrose-Orange G loading buffer (50 mM Bis–Tris propane pH 8.0, 40% w/v sucrose + Orange G dye. Samples were applied to a 0.5 × TBE, 5% v/v polyacrylamide gel (19:1 acrylamide: bis-acrylamide). Electrophoresis was carried out at 120 V for 35 min at 4°C, in 0.5× TBE. Separated species were visualized using a Fuji FLA-5100 Fluorescent Image Analyser.

## Results

### The iDDR motif of TRF2 interacts directly with RAD50

**Figure 1.**
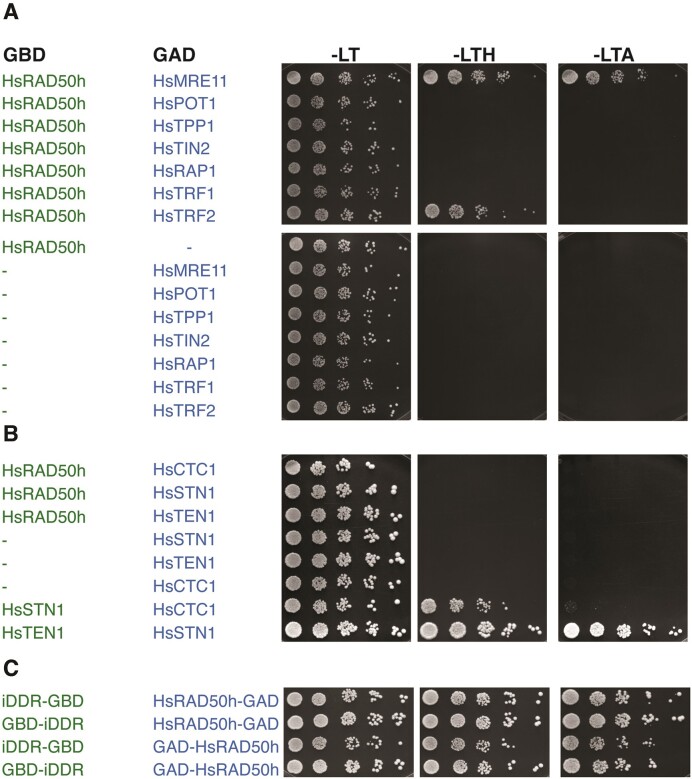
The iDDR motif of TRF2 binds RAD50. (**A**) Yeast two-hybrid analysis of full-length human shelterin subunits fused to the GAD domain of Gal4 for interaction against GBD-HsRAD50 encompassing HsRAD50 amino acids 1–221,1098–1312, separated by an SGSSAGG linker; this HsRAD50 ‘head’ (HsRAD50h) construct included the ATP binding cassette but lacked the majority of the coiled-coil ‘arm'. Culture dilutions were spotted on -LT plates (lacking tryptophan and leucine) as controls for cell numbers, -LTH plates (lacking tryptophan, leucine and histidine) for assessment of interaction, and -LTA plates (lacking tryptophan, leucine and adenine) for more stringent assessment of interaction. HsMRE11 was used as a positive control for interaction with HsRAD50h. (**B**) Analysis as in (A) testing the interaction of the CST subunits HsCTC1, HsSTN1 and HsTEN1 with the HsRAD50h construct. (**C**) Analysis as in (A) testing the interaction of the HsTRF2 region spanning amino acids 449–473, which includes iDDR, fused to the GBD of Gal4, either at the N- or C-terminus, with the head domain of human RAD50 (HsRAD50h) fused to either the N- or C-terminus of the Gal4 GAD domain; in all cases, constructs are named to indicate the relative positioning of the domains left-to-right referring to N-terminus to C-terminus. Negative controls (*vs* empty vectors) for both the wild-type TRF2 and RAD50 truncations were performed and did not show growth on selective -LTH and -LTA plates.

We recently reported identification of a short amino acid motif (MIN, for MRN inhibitor) in yeast telomeric proteins that can bind the RAD50 subunit of the MRN complex and disable the complex's function. There, we suggested that the MIN motif has evolved at least twice during evolution: in Orc4 in the Saccharomycotina, giving rise to the *Saccharomyces cereviase* (Sc) Rif2 MIN motif, and in the *Schizosaccharomyces* genus of the Taphrynomicotina, giving rise to the *Schizosaccharomyces pombe* (Sp) Taz1 MIN motif (37). This identified a potential general vulnerability in MRN and pointed to the possibility that other organisms might have independently evolved the same functionality within their telomeric proteins to regulate the activity of MRN, conceivably though the use of different motifs with unrelated primary sequence. Since we failed to identify the MIN motif in interesting candidates in the human genome by sequence analysis, we decided to investigate the ability of human core telomeric proteins—i.e. members of the shelterin complex—to interact with RAD50, using a yeast two-hybrid approach (Y2H). Our Y2H data revealed that TRF2 is unique among shelterin proteins in its ability to bind RAD50 (Figure 1A), suggesting a direct interaction, given that yeast is a heterologous system for human proteins; this conclusion is in agreement with previous work showing association of TRF2 with MRN and a preferential enrichment of the RAD50 subunit, over MRE11 and NBS1, in TRF2 immunoprecipitates (49,59). Moreover, no interaction with RAD50 was observed for proteins of the CST (CTC1, STN1, TEN1) complex (Figure 1B). As previous work had attributed the ability of TRF2 to bind MRN to the short iDDR motif in the hinge region of TRF2—at least when inserted in full-length TRF1 (49)—we checked the ability of iDDR to bind RAD50 on its own. The iDDR motif, spanning TRF2 amino acids (aa) 449–473, was found to be sufficient for interaction with RAD50 in two configurations tested, specifically when it was fused to the GBD motif of Gal4 at either its N- or C-terminus (Figure 1C). Taken together, our results suggest that the iDDR motif of TRF2 is the only region in the core telomeric complex capable of interacting with the ATP-binding globular region of RAD50.

### The iDDR motif binds to the regulatory interface of RAD50 also recognized by the MIN motif

**Figure 2.**
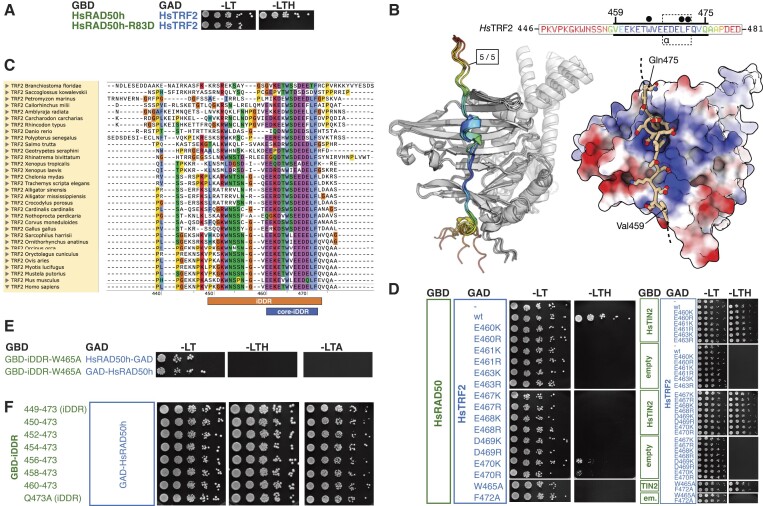
A model for binding of the iDDR motif to RAD50. (**A**) Yeast two-hybrid analysis of full-length HsTRF2 fused to the GAD domain of Gal4 for interaction against GBD-HsRAD50 encompassing HsRAD50 amino acids (aa) 1–221,1098–1312 either in wild-type form or carrying a R83D mutation. (**B**) Left: superposition of five independent AlphaFold2 models for the head domain of human RAD50 in complex with the iDDR of HsTRF2. Models are shown in cartoon representation, where HsRAD50 is coloured in grey and the identified interacting region of HsTRF2 coloured according to pLDDT score, using a continuous ‘rainbow’ spectrum from red (low confidence) to blue (high confidence). The amino sequence for the displayed section of HsTRF2 (aa 446–481) is provided as an inset. The thick black bars indicate the portion of sequence included in the model displayed on the right. An interacting region of HsTRF2 comprising amino acids Gly458-Gln475 was predicted with high confidence and consistency, containing a short helix segment spanning amino acids 468–472 (see inset: indicated with a dotted outline and labelled ‘α’). Right: molecular surface of HsRAD50 coloured with respect to electrostatic potential. The top-ranked model for the interacting region of HsTRF2 is shown in stick-representation, with backbone carbon atoms coloured in ‘light tan’. This visualisation reveals a high degree of charge complementarity between the two interacting proteins. Amino acids residues Trp465, Leu471 and Phe472 of TRF2 coalesce to form a hydrophobic ‘plug’ that inserts itself into a small receiving pocket found on the surface of the HsRAD50 head domain (indicated by filled black circles above the primary sequence). (**C**) Multiple amino acid sequence alignment for putative iDDR regions from the indicated species, executed and displayed using the Clustal Omega functionality of SnapGene v.6. The annotation and numbering provided at the bottom of the alignment corresponds to the amino acid sequence of the human protein. (**D**) Yeast two-hybrid analysis as in (A) of full-length HsTRF2 variants carrying the indicated mutations. All HsTRF2 variants were also tested against full-length HsTIN2 fusions to confirm protein functionality. (**E, F**) Yeast two-hybrid analysis as in (A) of the HsTRF2 iDDR (defined here as HsTRF2 amino acids 449–473, in wild-type from or carrying point mutations or N-terminal deletions, as indicated.

Previous analyses for binding of the yeast MIN motif to RAD50 pointed to an interaction with one surface of a β-sheet located in the N-terminal half of the globular head domain of RAD50 (37,38,41). We refer to this as the ’S’ region of RAD50, from the name previously given to the class of *S. cerevisiae* Rad50 alleles originally identified on the basis of their meiotic phenotype (60). The archetypal *rad50-S* allele is rad50-K81I, which results in loss of interaction with both Sae2 and the Rif2 MIN motif (37,38,41,61). We therefore mutated the corresponding arginine residue in human RAD50 to aspartic acid and tested the ability of this mutant (R83D) to interact with TRF2. RAD50-R83D failed to bind TRF2 (Figure 2A) suggesting that the iDDR, like the MIN motif in yeast, binds to the S region of RAD50.

**Figure 3.**
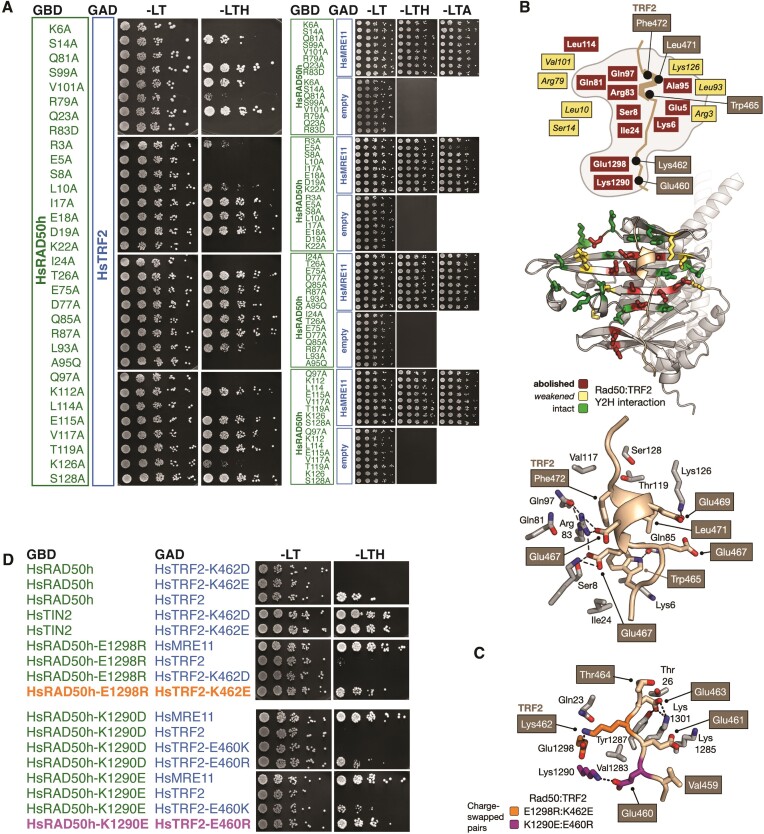
Map of RAD50 binding to iDDR. (**A**) Yeast two-hybrid analysis of full-length HsTRF2 fused to the GAD domain of Gal4 for interaction against GBD-HsRAD50 encompassing HsRAD50 amino acids 1–221,1098–1312 either in wild-type form or carrying the indicated mutations. In all cases, the HsRAD50 variants were tested against full-length HsMRE11 to confirm protein functionality, and with empty vector to rule out self-activation. (**B**) Top and middle: schematic overview and molecular cartoon for interactions mapped by Y2H. The side chains for each mutation tested are shown in stick representation, coloured with respect to their effect on HsTRF2 interaction: red = interaction is abolished, yellow = interaction is weakened, green = interaction is intact / unaffected. Bottom: additional molecular cartoon, providing additional detail for the mapped yeast 2-hybrid data, showing amino acids in close proximity to the modelled TRF2-helix. Here and in (**C**), predicted hydrogen-bonds are represented by black dotted lines and elected amino acids of HsTRF2 are annotated in white text on a grey/fawn background. See provided key for additional detail. (C) Molecular cartoon showing details for the amino acid charge-swap experiment shown in (D). (**D**) Y2H assay as in (A) of indicated full-length TRF2 and RAD50 ‘head’ variants. The two mutant pairings resulting in a rescue of the interaction defects of the single mutants are indicated in purple and orange and match the colouring in (C).

To test this idea further, we used the multimer mode of AlphaFold2 using ColabFold (56) to generate potential models for the iDDR motif in complex with RAD50. Each of the five resultant models had high confidence scores and a consistent mode of interaction (Figure 2B). The predicted RAD50-interacting region within the iDDR motif runs approximately from aa 459 to 475 of TRF2 and is arranged perpendicularly to the strands forming the β-sheet in RAD50. Reassuringly, Arg83 makes a specific contact with Asp469 of TRF2 in the model (see below, Figure 3B). Interestingly, aa 460–472 are also the most strongly conserved positions within the iDDR itself (Figure 2C), and for simplicity we will refer to this region here as the ‘core-iDDR’ motif. In the AlphaFold2-model, the conserved region contains a run of charged amino acids positioned to interact with side chains of RAD50 carrying an opposing charge (Figure 2B, right). We tested the relevance of these interactions in yeast two-hybrid assays, by use of several charge-swap mutations: each of the charged amino acid residues within TRF2 region 461–470 tested were found to be important for interaction, with only Glu470 retaining some residual activity in the binding assay (Figure 2D). Interaction with TIN2 was used as a control for functionality of the TRF2 variants. We also confirmed the importance of the two single most conserved residues within the iDDR, Trp465 and Phe472: changes to alanine at either of these positions abrogated the ability of TRF2 to bind RAD50 (Figure 2D), in agreement with them coming together with Leu471 to form a hydrophobic bundle or ‘plug’ that interacts with a pocket on the surface of RAD50 (see below). The Trp465 mutation also abolished binding in the context of the isolated iDDR, even though binding of the domain alone appears stronger (in wild-type form) than full-length TRF2 in Y2H (as the interaction is more apparent under stringent selection on medium lacking adenine; compare Figure 1A and C). Because no interaction of TRF2 with RAD50 was predicted by the model outside of the core-iDDR motif, we tested several iDDR deletions by Y2H, confirming that the motif in isolation was sufficient for interaction (Figure 2E). Together, these results indicate that the most conserved region of iDDR is sufficient for association with RAD50 and that the majority of the residues within this region are required for binding, underscoring a high degree of specificity.

We further validated the binding model by mutating a large set of residues within the S region of RAD50, selecting for analysis those residues with side chains protruding towards the surface of the protein, and assessing their ability to interact with TRF2 by Y2H assay (Figure 3A). This analysis yielded data consistent with the AlphaFold2 model, with mutations that reduced or ablated the interaction mapping, with close proximity, to the predicted position of the core-iDDR (Figure 3B, top and middle). The hydrophobic grouping of TRF2 residues Trp465, Leu471 and Phe472 is positioned directly above Ala95 of RAD50: a change of this residue to a bulkier glutamine side chain was found to disrupt binding. Additional mutations in the S region of RAD50, both to the left and to the right of the modelled C-terminal segment of iDDR, were also found to either ablate or partially reduce binding.

To test the AlphaFold prediction further, we interrogated the model to identify charged interactions between amino acid pairs in the two proteins that could lead to loss of binding when mutated in isolation, but where restoration of binding might be expected if the two charged residues were swapped. For this, we focussed on the N-terminal region of the core-iDDR, which is predicted to interact largely with the C-terminal ATP-binding domain of RAD50 (Figures 2B and 3C). We identified Lys462 in iDDR, and Glu1228 and Lys1290 in RAD50 which when mutated to a residue of opposite charge (Asp/Glu at 462, Arg at 1228 and Asp/Glu at 1290) lead to a loss of binding in the Y2H assay (Figure 3D). However, when the Glu460Arg mutation (previously shown to destroy binding; Figure 2B) was combined with a Lys1290Glu mutation, the interaction defect of the individual mutants was fully rescued. A similar effect was observed for the Lys462Glu / Glu1298Arg pairing (Figure 3D).

Taken together these results provide direct experimental support for a specific interaction of the iDDR motif with a regulatory region within RAD50, thus suggesting that TRF2 might employ a similar mechanism for MRN inactivation as that enforced by the MIN motif in yeast.

### The MIN motifs of Rif2 and Taz1 bind RAD50 at a position similar to that of the iDDR motif

**Figure 4.**
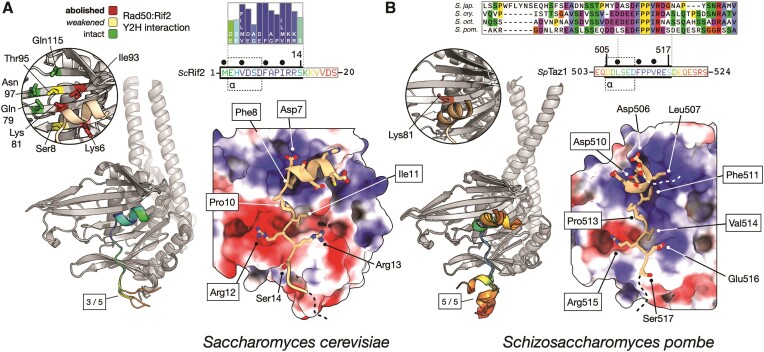
Models for ScRif2 and SpTaz1 MIN interactions with Rad50. (**A**) Left: superposition of three independent AlphaFold2 models for the head domain of ScRad50 in complex with the MIN motif of ScRif2. Models are shown in cartoon representation, where ScRad50 is coloured in grey and the identified interacting region of ScRif2 coloured according to pLDDT score using a continuous ‘rainbow’ spectrum from red (low confidence) to blue (high confidence. The primary sequence for the first 20 amino acids of ScRif2, including the MIN motif, is provided as an inset, using the same pLDDT colour scheme and aligned to the MIN consensus derived from Orc4 and Rif2 sequences (37). Thick black bars indicate the portion of sequence included in the model displayed on the right. A region comprising amino acids 1–15 was predicted with high confidence and consistency, containing a short helix segment spanning amino acids 2–7 (see inset: indicated with a dotted outline and labelled ‘α’). The circular inset, shown on the left maps the results of mutational analysis examining binding of ScRif2 to ScRad50 coloured as per Figure 3B (38). Right: molecular surface of ScRad50 coloured with respect to electrostatic potential. The top-ranked model for the interacting region of ScRif2 is shown in stick-representation, with backbone carbon atoms coloured in ‘light tan’. This visualisation reveals a high degree of charge complementarity between the two interacting proteins. Amino acids of ScRif2, including Phe8, that make fully buried hydrophobic interactions with the surface of ScRad50 are annotated above the primary sequence inset, as shown by filled black circles. The five core positions in the MIN consensus from the Saccharomycotina [ScRif2 7–12 and SpTaz1 508–513] which allowed identification of MIN in SpTaz1 by homology search (37) are bounded by a black rectangle in the annotation in both panels. (**B**) Predicted interaction between the SpTaz1 MIN motif and SpRad50, using the same representations as those shown in panel (A). A Clustal Omega alignment of the four Schizossacharomyces Taz1 amino acid sequences is provided as an inset. A region spanning amino acids 505–517 was predicted with high confidence and consistency, containing a short helix segment comprising amino acids 506–510 (see inset: indicated with a dotted outline and labelled ‘α’). The secondary inset, highlights the location of Lys81, whose mutation prevents binding of SpTaz1 to SpRad50.

Because the MIN and iDDR motifs lack any amino acid sequence similarity but share a common binding partner in RAD50—which in eukaryotes is highly conserved in amino acid sequence particularly in the globular region—we also modelled binding of the ScRif2 and SpTaz1 MIN motifs to their respective RAD50 partners, to assess any universality of this mechanism for MRN inactivation. AlphaFold2 predicted interactions in both cases; again, like for iDDR, the regions of high confidence in the models corresponded with the most conserved regions of both motifs (Figure 4A and B). In both the *S. cerevisiae* and *S. pombe* models, the N-terminal section of the MIN motif adopted an alpha helical structure, that bound in close proximity to the side chain of Rad50-Lys81. Pleasingly, this amino acid is required for interaction in both species (37,38,41,61). In addition, the ScRif2 model is remarkably consistent (Figure 4A) with a previous mutational analysis carried out for budding yeast RAD50 (38). We note, however, that asparagine 18, located to the far left of the RAD50 β-sheet was also shown to affect interaction with Rif2 when mutated to serine (41). We speculate that this mutation might affect interaction with the more C-terminal section of the Rif2 MIN motif, not accounted for in our model, but required for binding in yeast two-hybrid assays (38). For the two MIN models, there is a remarkable degree of similarity in the interactions made by the central part of each motif, with Phe8/Phe511 of RIF2/Taz1, respectively, sitting in a small pocket on the surface of Rad50, along with identical positions of residues Pro10/Pro513, and Arg13/Arg515 as part of a larger phenylalanine-hydrophobic-proline-hydrophobic-arginine (FφPφR) motif (Figure 4A and B). In both cases, a short, predominantly negatively-charged helical element precedes the FφPφR motif, contributing to the binding interface through interaction with a reciprocal, positively charged Rad50 surface. The electrostatic charge distribution of the Rad50 S regions in the two yeasts is, however, quite different across the coiled-coil-distal part of each region (as illustrated in Figure 4), with ScRad50 having a more pronounced negatively charged surface, which matches the propensity of the ScMIN motif to bear positively charged residues near the C-terminal end of the minimal sequence (Figure 4A) (37), whereas the SpMIN at the equivalent positions contains negatively charged resides (Figure 4B).

In conclusion, our structural predictions for each RAD50-MIN interaction are consistent with published mutational analyses that assess binding capability, and display an overall similarity to the predicted binding position of the iDDR on RAD50, suggesting that these telomeric factors exploit a common mechanism to inactivate MRN and pointing to a shared mechanism of action.

### The RAD50 surface for interaction with CtIP/Sae2/Ctp1 is predicted to partly overlap with that for the iDDR/MIN motifs

**Figure 5.**
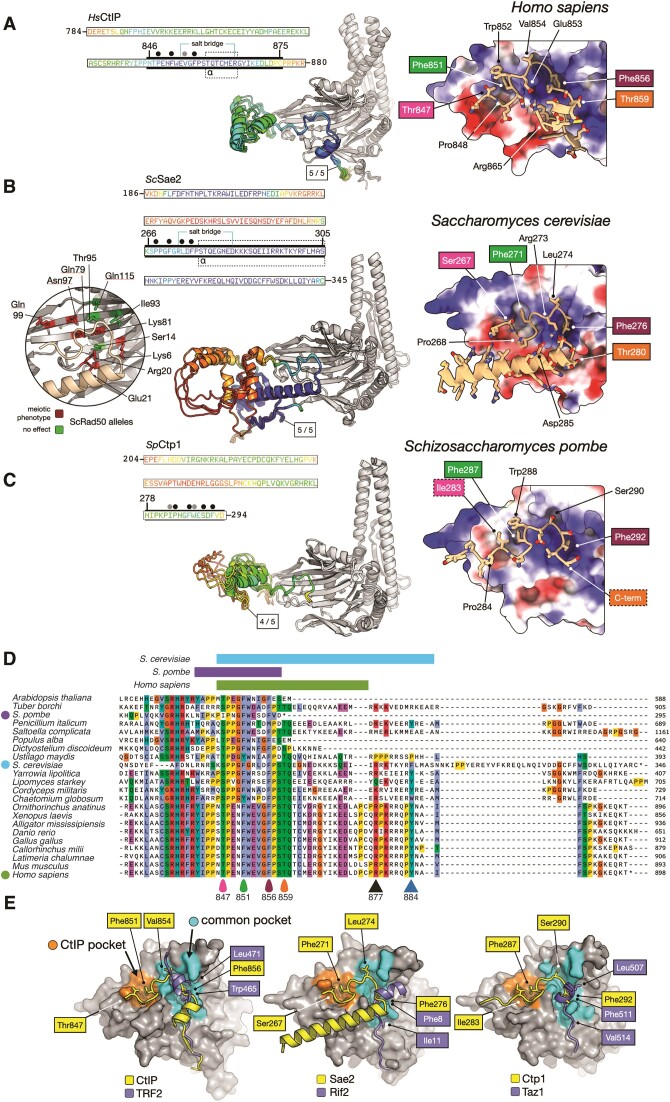
Models for HsCtIP, ScSae2 and SpCtp1 interactions with Rad50. **(A**–**C)** Left: the number of AlphaFold2 models (out of the 5 generated) with consistent binding pose are indicated, shown in cartoon representation, and coloured according to pLDDT score using a continuous ‘rainbow’ spectrum from red (low confidence) to blue (high confidence). The head domain of RAD50 is also shown in cartoon representation and coloured grey. In each case, the amino acid sequence for the region predicted with high-to-medium confidence is provided as an inset. Thick black bars indicate the portion of sequence included in the figure displayed to the right. Right: Molecular surface of RAD50 coloured according to electrostatic potential, with the top-ranked model shown in combined cartoon / stick representation, and backbone carbon atoms coloured in ‘light tan’. (A) HsRAD50 in complex with CtIP. A region comprising amino acids Gln791-Asp873 was predicted with high confidence and consistency, containing a short helix segment spanning amino acids Thr859-Arg865 (see inset: indicated with a dotted outline and labelled ‘α’). The model also suggests formation of an internal HsCtIP salt-bridge between the side chains of Glu853 and Arg865. Right: This visualisation reveals a high degree of charge complementarity between the two interacting proteins, with HsCTIP residues Pro848, Phe851, Val854 and Phe856 making additional hydrophobic interactions with the surface of the HsRAD50 head domain (highlighted by black filled circles placed above the inset primary sequence if fully buried, or dark grey if packed against the surface). Selected amino acid side chains are shown in stick representation, with labels coloured according to the ‘teardrops’ placed below the multiple amino acid sequence alignment shown in panel (**D**) for a few conserved residues, including HsCTIP-Thr847 as a known site of phosphorylation. (B) ScRad50 in complex with ScSae2. A region spanning amino acids Ser265-Cys345 was predicted with high confidence and consistency, containing a helix segment spanning amino acids Lys266-Ser305 (see inset: indicated with a dotted outline and labelled ‘α’). The model also suggests formation of an internal ScSae2 salt-bridge between the side chains of Arg273 and Asp285. Residues Pro268, Phe271, Leu274 and Phe276 of ScSae2 make buried hydrophobic interactions with the surface of the ScRad50 head domain (highlighted by black filled circles placed above the inset primary sequence). The circular inset summarizes genetic data for the possible interaction of ScSae2 with ScRad50 (38,60). Right: selected amino acid side chains are shown in stick representation, with labels coloured according to the ‘teardrops’ placed above the multiple amino acid sequence alignment shown in panel (D) for a few conserved residues, including ScSae2-Ser267 as a known site of phosphorylation. (C) SpRad50 in complex with SpCtp1. A region spanning amino acids Gln265-Val293 was predicted with medium confidence and consistency. Residues Ile283, Pro284, Phe287, Trp288, Ser290 and Phe292 of SpCtp1 make hydrophobic interactions with the surface of the SpRad50 head domain (highlighted by black filled circles placed above the inset primary sequence if fully buried, or dark grey if packed against the surface). Right: selected amino acid side chains are shown in stick representation, with labels coloured according to the ‘teardrops’ placed above the multiple amino acid sequence alignment shown in panel (D) for a few conserved residues. (**D**) Multiple amino acid sequence alignment for the C-terminal region of HsCtIP/ScSae2/SpCtp1, plus homologues from the indicated species, executed and displayed using the Clustal Omega functionality of SnapGene v.6. The three coloured bars above the alignments refer to the sequence marked by the thick black bars in (A-C), indicating the portion included in the structural models. Teardrops at the bottom of the alignments indicate conserved phosphorylation sites and aromatic residues, also highlighted in the models in (C); the triangles indicate conserved residues, mutated and described in Figure 6, and belonging to the ‘extended interface’ of CtIP. (**E**) Schematic summary of AlphaFold2-modelling for the interaction of each indicated motif, with the surface of RAD50 (coloured grey). iDDR/MIN motifs predominantly interact with a singular pocket found on the surface of RAD50 (coloured cyan, and labelled ‘Common pocket). The CtIP orthologues interact additionally with a second pocket (coloured orange, and labelled ‘CtIP pocket’). Selected amino acids residues are labelled for reference.

It was previously shown that the MIN motif of Rif2 impairs the endonucleolytic activity of MRN *in vitro*, as well as resection near DSBs when the MIN motif is tethered near a break site (37,38). In addition, Rif2 was shown to partly suppress the DNA repair defect of *sae2* mutants (41). This has lead to the idea that the MIN motif might act to inhibit MRN nuclease action by preventing the association of the CtIP/Sae2/Ctp1 cofactor (41), which is required for DNA cleavage (11–16). We therefore decided to investigate the binding mode of the three orthologues CtIP, Sae2 and Ctp1 to their respective RAD50 counterparts using AlphaFold2 as before. This analysis predicted interaction with the S region of RAD50 for all three proteins utilizing the conserved C-terminal ‘Sae2-like’ region (62) (Figure 5A-C). Pleasingly, the model for ScSae2 agrees with the available genetic data and the distribution of the original *rad50-S* alleles (Figure 5B) (38,60). These models for MIN/iDDR and Sae2/Ctp1/CtIP binding offer a mechanistic explanation for the proven abilities of Rif2 and TRF2 to inhibit Sae2- and CtIP-dependent endonucleolytic activity of MRX/MRN (37,41,52).

Here, the three models identify two main conserved areas, or ‘pockets’, for CtIP/Sae2/Ctp1 interaction with RAD50 (Figure 5E): a ‘common pocket’ that largely overlaps with the binding site for the iDDR/MIN motifs as described above, whereas the ‘CtIP pocket’ is located to its left (as represented in Figure 5E) in a part of the β-sheet region where our modelling did not identify strong iDDR/MIN binding. The predictions uncover similarities in the binding mode of CtIP/Sae2/Ctp1 to RAD50, placing recognised critical phosphorylation sites in Sae2 and CtIP (Ser267 and Thr847, respectively) for interaction in the ‘CtIP pocket’ (15,61,63,64). Furthermore, the RAD50-interacting portion of SpCtp1 is restricted to the last 17 aa in the protein and almost exactly coincides with a C-terminal 15-aa peptide that is capable of stimulating MRN nucleolytic activity *in vitro* (65). Notably, the MIN and iDDR motifs and the Sae2-like domains all bear hydrophobic amino acids which are among the most highly invariant in each motif/domain and which make deep contacts into a conserved small cavity in RAD50: these are Phe8 in the ScMIN; Phe511 in the SpMIN; Trp465, Leu471 and Phe472 in the HsiDDR; Phe865, 276 and 292 in the HsCtIP, ScSae2 and SpCtp1 Sae2-like domains, respectively (Figures 2B, 3B, 4).

Our Y2H-data, plus that of others, combined with interaction modelling by AlphaFold2, suggest a pattern of interactions that identify a conserved mode of interaction for MRN co-factors in binding to RAD50, which is consistent with the notion that iDDR or MIN binding is mutually exclusive to that of CtIP/Sae2/Ctp1.

### CtIP has an extended RAD50 binding interface

**Figure 6.**
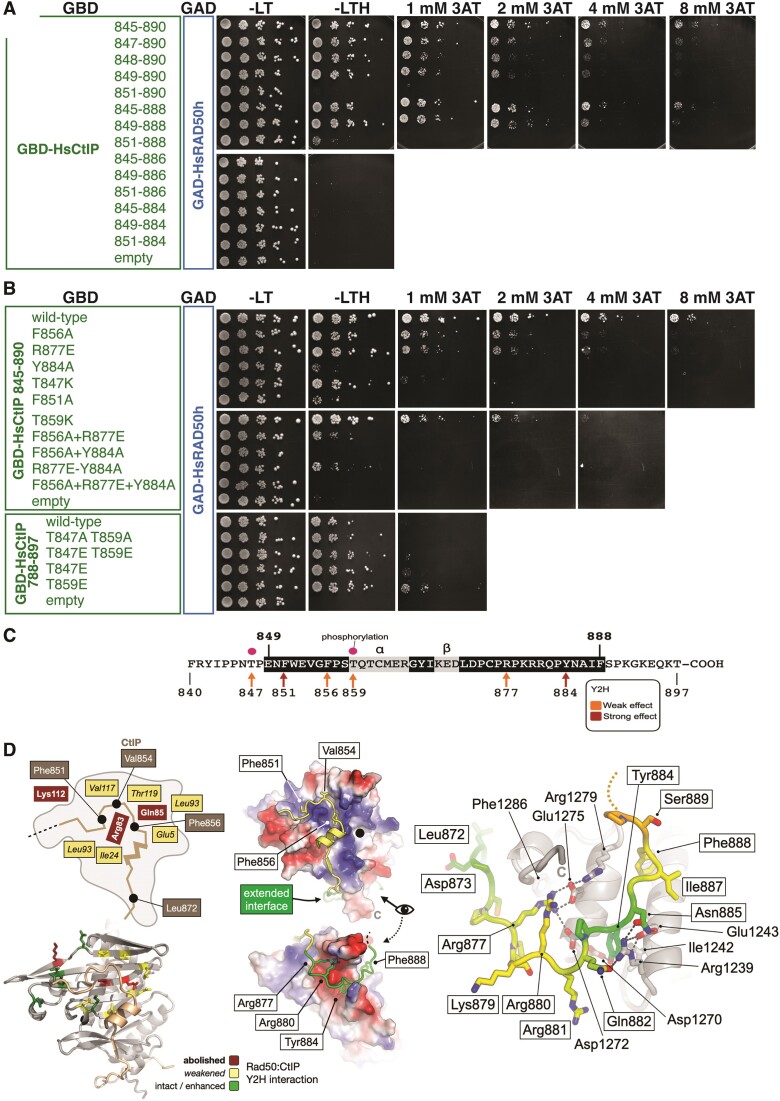
Characterization of the amino acid sequence requirements for binding of CtIP to RAD50. (**A**) Y2H analysis of portions of HsCtIP containing the indicated amino acids fused to the GBD domain of Gal4 for interaction against GAD-HsRAD50 ‘head’. The stringency of the selection on plates lacking histidine was increased by the addition of the indicated concentration of 3-amino-1,2,4-triazole (3AT). (**B**) Y2H analysis of the indicated CtIP mutations, made in the context of a GBD fusion to amino acids 845–890 or 788–897, as indicated, of CtIP. (**C**) Schematic summarizing data obtained from the Y2H experiments presented in panel A and B and in Supplementary Figures S1–S5. Amino acids 849 to 888 are necessary and sufficient for interaction between RAD50h and CtIP, this incorporates an alpha-helix spanning Thr859 to Arg865 and a short beta-strand from Lys868 to Asp870 (labelled α and β respectively, and indicated by black text on a grey background). The effect of mutations at various sites are colour-coded, with an orange colour indicating that the effect of a mutation at this site is relatively weaker, and red that the effect is relatively stronger (a weak effect being detectable in double mutants and/or at higher stringency). The known sites of phosphorylation at Thr847 and Thr859 are highlighted (purple circles). See provided key for additional information. (**D**) Left: summary of Y2H data, shown as a schematic (top) and molecular cartoon (bottom). Selected amino acids are shown in stick representation, and the effect of their mutation shown by a red/orange/green traffic light colour scheme, indicating loss, weakened, and unperturbed/enhanced interaction respectively. Middle: molecular surface of RAD50 coloured by electrostatic potential, overlaid with a cartoon representation of the model for an extended CTIP interface (coloured green). Right: molecular cartoon presenting details for the extended interface. Selected amino acid side chains are shown in stick representation, coloured by pLDDT score using a continuous ‘rainbow’ spectrum from red (low confidence) to blue (high confidence). Predicted hydrogen-bonds are indicated by black dotted lines.

To confirm our predictions, we tested several CtIP fragments for binding to RAD50 by Y2H. This analysis showed that the minimal CtIP region required for binding to RAD50 spans amino acids 849–888 (Figure 6A) and includes the stretch predicted with the highest confidence as a RAD50 interactor (aa 846–875, Figure 5A, D) and it also revealed that that this minimal interacting region also includes additional residues in the C-terminal portion of CtIP predicted with lower confidence by AlphaFold2 (we refer to this region as the ‘extended interface’, see Figure 6D).

To gain further experimental support for the structural predictions, we engineered specific mutations within the minimal RAD50 interacting domain of CtIP. Analysis of CtIP orthologues across several species identified strongly conserved aromatic residues, in particular Phe851 and Phe856: mutation of Phe851 to Ala had a strong negative effect on the ability of a fragment of CtIP spanning aa 845–890 to bind RAD50, whereas a Phe856 mutation had a smaller defect but which was clearly detectable under conditions of higher stringency (Figure 6B). We then also tested two mutations (both to alanine) within the ‘extended interface’ of CtIP, Arg877 and Tyr884, as they are predicted to make electrostatic interactions with Glu1275 and Asp1272, and fit into a pocket formed between two alpha helices of RAD50, respectively (Figure 6D). In support of the model, the Tyr884Ala mutation had a strong negative effect on the interaction, with Arg877Ala also dampening it, albeit to a lesser extent (Figure 6B). As expected, combining the two weaker mutations Phe856Ala and Arg877Ala had a strong additive effect (see plating in 1 mM 3AT, Figure 6B).

This mode of CtIP binding was tested further by assessing the ability of the 845–890 CtIP fragment to bind to a large panel of RAD50 mutants, as done previously for the iDDR (Figure S1). None of the RAD50 mutations tested was found to eliminate growth on plates lacking histidine, but clear defects could be detected on higher-stringency plates, where 4 mM 3AT was employed. Under these conditions a spectrum of defects was detected, with Arg83Glu, Gln85Ala and Lys112Ala having the strongest effect on binding, and Glu5Ala, Asp19Ala, Ile24Ala, Leu93Ala, Gln97Ala, Val117Ala and Thr119Ala having a milder effect (Figure S1, Table S1, Figure 6E). Given the relatively moderate effect of many of the RAD50 mutations against wild-type, we decided to test their effects in the context of similarly mild counterpart variants of CtIP. The results with the pairs of mutants corroborated the importance of the role of the RAD50 residues identified above (Figures S2-5, Table S1). Interestingly, mutation to Ala of the two threonines (847 and 859) which are known to be *in vivo* phosphorylation sites important for CtIP function, was not found to affect RAD50 binding compared to the wild-type counterpart, but a phosphomimic mutation at 859 marginally improved binding (Figure 6B). Similarly, a defect for the Thr847Lys and Thr859Lys variants was only uncovered at higher stringency and/or in the context of several RAD50 mutant backgrounds (Figures S4 and S5, Table S1), revealing that alterations at these known CtIP phosphorylation sites weaken the overall affinity of CtIP for RAD50. Overall, these results are consistent with the existence of a relatively large interaction interface in CtIP, with several residues from both RAD50 and CtIP making incremental contributions to binding.

### The iDDR motif disrupts binding of RAD50 to DNA

**Figure 7.**
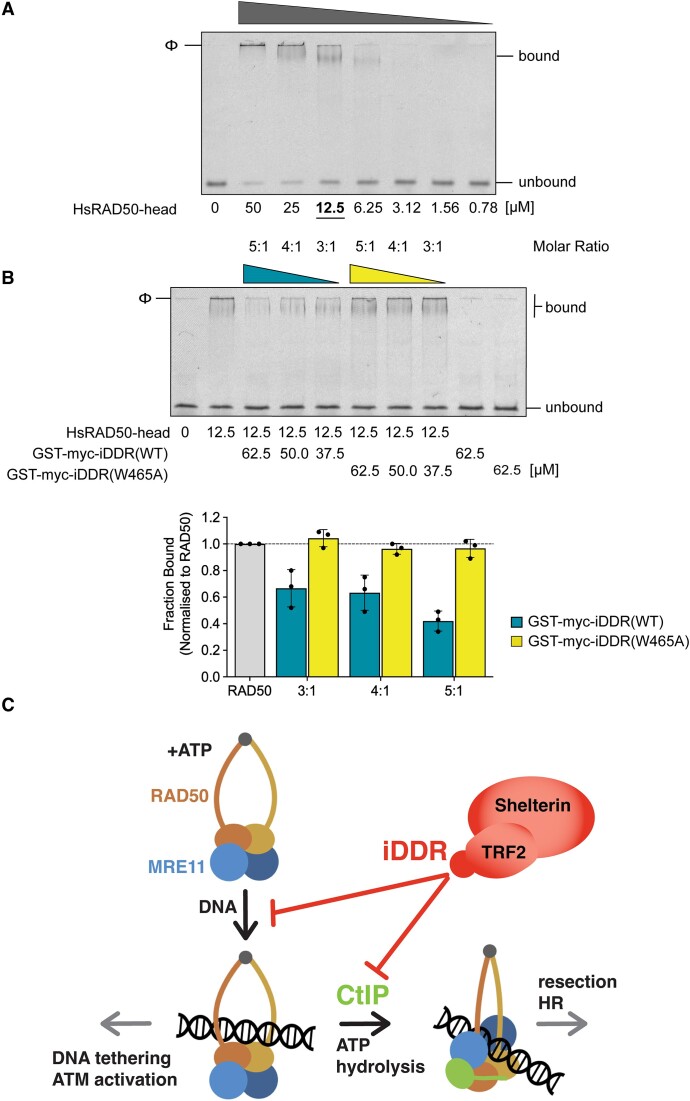
The iDDR perturbs DNA-binding by RAD50. (**A**) Electrophoretic-mobility shift assay (EMSA) demonstrating interaction of purified recombinant HsRAD50-head, in the presence of ATP, with a fluorescently-labelled 48mer DNA duplex. (**B**) Competition assay. (Top) Addition of iDDR(WT) perturbs the ability of HsRAD50 to bind DNA. Mutation of Trp465 to alanine (W465A) abrogates this effect. (Bottom) Quantitation of replicate EMSA experiments (three individual experiments). Error bars represent one standard deviation. (**C**) Model summarizing the proposed dual role of the iDDR in controlling MRN function at telomeres: first, in preventing CtIP binding to RAD50 and therefore activating MRE11 nuclease activity and, second, in inhibiting DNA binding by RAD50, which could affect the tethering and ATM-activating functions of MRN.

The interaction of CtIP with MRN serves to activate the nuclease function of MRE11, which the MIN and iDDR motifs are well poised to counteract, as shown by an array of *in vitro* and *in vivo* experiments (37,41,52). However, we and others have previously shown that the MIN motif of Rif2 also prevents NHEJ and Tel1 activation, neither of which relies on Sae2/Mre11 nuclease action (37,41,52). How the MIN motif carries out this additional function is not currently clear, but might involve stimulating the ATPase activity of RAD50 and thus have an effect on its allosteric transitions, and/or inhibiting its DNA-binding activity (38,40,45). Therefore, we decided to test whether the iDDR might have an effect on the ability of RAD50 to bind DNA. To this end, we performed electrophoretic mobility shift assays (EMSAs) with purified recombinant ATP-binding globular RAD50 domain (HsRAD50-head) and a fluorescently-labelled 48-mer double-stranded linear DNA. As expected, we could detect a RAD50-DNA complex in the gels in the presence of ATP (Figure 7A). Strikingly, the addition of increasing amounts of purified recombinant iDDR domain (fused to GST) showed a clear negative effect on the ability of RAD50 to bind DNA in this assay, which was correlated to the amount of the iDDR protein present (Figure 7B). This property of iDDR to inhibit DNA binding by RAD50 was related to its ability to associate with the protein, as an iDDR variant carrying the Trp465Ala mutation, which disrupts association to RAD50 in the Y2H assay, was defective in this activity (Figure 7B). Similar results were obtained when the iDDR was fused to an alternative solubilization domain, thioredoxin (TRX; Figure S6), confirming a specific effect of the iDDR motif. Taken together, these results indicate that the iDDR has a multi-faceted effect on MRN activity, that is not simply limited to the control of CtIP association with the complex.

## Discussion

Our previous work in budding and fission yeast uncovered an amino acid motif dedicated to inhibition of MRN activity, which we proposed independently arose in DNA replication origin factor Orc4 (and then, through sub-functionalization, was retained by telomeric protein Rif2) and in telomeric protein Taz1 (37). We called this motif ‘MIN’, for MRN inhibitor, to highlight its potency in disabling the multiple facets of MRN complex activity. Here we provide structural modelling, obtained using AlphaFold2, that provides details for the most conserved section of the Rif2-RAD50 interaction, and which is consistent with previous mutational data examining their interaction (38). Strikingly, a model for interaction of the MIN motif from SpTaz1 with SpRad50 predicts a similar set of interactions. In particular, several of the contacts made are highly similar between the two organisms, and closely correspond to the most conserved amino acids residues in a ‘pile-up’ compiled from Orc4 and Rif2 MIN motifs in the Saccharomycotina (37), explaining why we were previously able to identify the MIN motif in the *Schizosaccharomyces* species based on amino acid sequence identity alone. However, changes in the electrostatic surface potential of the RAD50 S region, which are apparent in the three structures presented here, would by necessity lead to changes in the amino acid sequence composition of a MIN-like peptide, and therefore similar short RAD50-binding motifs/activities might be easily missed in analyses by protein sequence alone. For example, the MIN-motifs of Rif2 and Taz1 share a general preponderance of negatively charged amino acids at their respective N-terminal ends, whereas the region more C-terminal to the core of the motif contains a swap in the charge of a single amino acid, i.e. the equivalent of Rif2-Arg13 is Taz1-Glu516, thus matching a concomitant change in electrostatic potential on the surface of Rad50.

The data we present here demonstrate that the iDDR motif of human TRF2 makes use of the same region when binding to RAD50. The evolution of telomere DNA binding proteins appears complex and is not well understood, but TRF2 is possibly not a direct orthologue of Taz1, given that the two proteins are structurally different (5,7,9,66). Regardless of any common ancestry, our analyses strongly indicates that the binding of the TRF2-iDDR motif to RAD50 represents a third example of evolution producing a RAD50 binding motif within a telomeric protein. The binding-position of both iDDR and MIN motifs on the surface of RAD50 is highly similar, in both cases containing a short alpha-helix that binds at the same location on Rad50, flanked by a patch of amino acids complementary to the charge present on the corresponding interaction surface of RAD50 (compare Figures 2B and 4A, B). However, the position of the charged stretch of amino acids is reversed in the two systems, with the alpha helix sitting at its C-terminal side in iDDR, but at the N-terminal side in the two MIN motifs. While in principle the different distribution of the charged amino acids in the iDDR and MIN could have diverged to match the electrostatic potential of RAD50, the observed inversion in polypeptide strand polarity, together with the difference in hydrophobic interactions—iDDR forming a distinctive hydrophobic ‘plug’ comprising amino acids Trp465, Leu471 and Phe472—demonstrate the that the iDDR/RAD50 interaction constitutes a third example of convergent evolution for this particular mechanism of RAD50 binding.

Our extensive mutational analysis of the iDDR motif provides direct experimental confirmation of our AlphaFold predictions and reveals that only the most highly conserved region of the iDDR motif is required for RAD50 association. This region spans amino acids 460–472 and, rather strikingly, all the changes we made (at 10 amino acid positions - three positions remain untested) disrupted binding, revealing a high degree of specificity. It presently remains unclear what function, if any, the amino acids N-terminal to the conserved iDDR core might be carrying out. It is of course possible that these residues could serve to increase the affinity of iDDR binding to RAD50, perhaps *in vivo* or in the context of the full MRN complex; we note that another group has included in a model additional RAD50-interacting residues from this part of iDDR, which are not present in our own current models (52). Alternatively, this portion of iDDR might play a more regulatory role in modulating MRN function; it will be interesting, for example, to investigate in more the detail the requirements for the inhibition of RAD50–DNA binding (see below).

ATP binding by RAD50 produces an allosteric switch in the MRN complex that suppresses endonuclease action, but is proficient for both DNA binding and kinase activation (17,42,43). The hydrolysis of ATP results in a repositioning of the MRE11 dimer from a position sitting ‘below’ the RAD50 head-domain in the ‘resting’ state, to a ‘up' or ‘sideways’ position compared to RAD50 in the ‘cutting’ conformation (Figure 7C) (44,67). Recent structural, biochemical and genetic studies, as well as modelling, indicate that CtIP/Sae2/Ctp1 might function to bridge a RAD50-MRE11 interface that, in the absence of the ‘fastener’ loop present in the bacterial orthologues of Mre11, stabilises the complex in its nuclease-proficient conformation, upon ATP hydrolysis (Figure 7C) (41,44,68). The combined genetic and biochemical analysis of Sae2 binding to Rad50 (38,60,61) pointed at the potential of a RAD50 β-sheet region to bind MRN regulators. Here we present structural modelling for the predicted mode of binding for CtIP/Sae2/Ctp1 to RAD50, which serve to validate earlier suggestions that the MIN motif might prevent endonucleolytic action by MRN by directly interfering with the ability of the required CtIP/Sae2/Ctp1 co-factor to bind RAD50 (37,38,41); we note that recent data has identified a role for the iDDR in limiting MRN-dependent resection at human telomeres and in inhibiting MRN CtIP-dependent endonuclease activity *in vitro* (52). Our data, presented here, offer experimental evidence in support of our AlphaFold predictions pointing to an expanded region of CtIP required for its binding to RAD50, with a conserved area (‘CtIP pocket’) that is seemingly reserved for CtIP binding, a central area (‘common pocket’, also conserved) that has extensive overlap with iDDR/MIN binding, and a C-terminal area (‘extended interface’) which is specific to human CtIP and is critical to stabilize binding. The analysis reveals that the similarities in binding among the CtIP orthologues are largely confined to a small region of each protein, coinciding with a region of high amino acid sequence conservation corresponding to amino acids 847–860 of HsCtIP, which overlaps with the ‘CtIP pocket’ and the ‘common pocket’. The picture that emerges is that of a relatively extended interaction surface for CtIP, partly overlapping with that of iDDR (as characterised in this study and coinciding with the conserved ‘core’ of the iDDR) but extending past it at both ends. Mutagenesis has revealed a relatively large degree of tolerance for individual mutations, with relatively minor and incremental effects on CtIP binding, sometimes apparent only in the context of combined mutations, which could be explained in principle by the length of the interaction interface (i.e. bipartite in nature).

Since the ‘common pocket’ in CtIP (Figure 5E) fully overlaps the iDDR/MIN binding site, CtIP/Sae2/Ctp1 and iDDR/MIN binding are predicted to be mutually exclusive. However, as parts of the extended MIN and iDDR motifs are only poorly modelled by AlphaFold2 (low pLDDT scores) it remains possible that any overlap between the binding interfaces is more extensive, as noted above, although this idea is not supported by our deletion analysis. These findings present a scenario of opposing regulatory activities exemplified by a positive regulator of MRN in the CtIP/Sae2/Ctp1 orthologues and a set of evolutionarily converged negative regulators in telomeric factors Rif2, Taz1 and TRF2. The interplay between these regulatory factors is, however, not clear and the relative affinities of each protein or interaction motif for RAD50 remain to be determined. However, due to its hydrophobic plug and thus larger buried surface area, the iDDR motif in particular might be able to bind RAD50 with high affinity. In addition, the local high concentration of telomeric factors are likely to shift the balance in favour of MRN inhibition specifically at chromosome ends.

The ‘CtIP pocket’ in RAD50 described above appears reserved for interaction with just CtIP/Sae2/Ctp1 and is conserved in its general geometry among these orthologues (Figure 5B). It is of note that the conservation in CtIP/Sae2/Ctp1 binding mode to RAD50 in the models is confined to this pocket and to the ‘common pocket’. This segment of each CtIP protein is also highly conserved at the sequence level (Figure 5D) and contains the phosphorylation sites critical to CtIP/Sae2/Ctp1 function in promoting resection (63–65,69). This part of CtIP has also been proposed to mediate an interaction with MRE11 instrumental to stabilising the active conformation of the MR complex, so this activating role might be at the heart of the structural similarities found among the orthologues within this region (68). We found that binding of human CtIP to RAD50 does not require prior phosphorylation at two conserved residues in the C-terminal domain (Thr847 and Thr859), unlike in yeast (61), as alanine substitutions at these positions do not abolish the interaction; more disruptive mutations (to Lys) did however affect the binding to some extent. These results are consistent with the idea that these phosphorylation events in human CtIP might have a role in the regulation and activation of the complex - rather than strictly in its assembly.

Immediately C-terminal to the segment making contacts across the two conserved ‘CtIP’ and ‘common’ pockets, amino acid residues from CTIP also contact a third area of human RAD50, with this extended binding mode in the human system highly divergent when compared to budding yeast and altogether absent in fission yeast (Ctp1 has a shortened C-terminus which therefore does not occupy this extended interaction area). We show that interactions in all three regions of the proposed CtIP-RAD50 interface (‘CtIP pocket’, ‘common pocket’ and ‘extended interface’), as exemplified by mutations in Phe851, Phe856 and Tyr884, are required for efficient docking of CtIP on RAD50 (Figure 5E,6D).

As discussed above, the iDDR/MIN motifs are expected to prevent CtIP/Sae2/Ctp1 binding, and therefore stabilization of the ‘cutting state’ of MRN. In budding yeast, Rif2 has been shown to increase the ATPase activity of RAD50 through the action of the MIN motif and this particular property has been invoked as being instrumental in mediating the effect of Rif2 in suppressing NHEJ and ATM/Tel1 activation at telomeres (37,38,40,45). While this scenario is attractive, it is presently not clear whether the iDDR motif might have a similar effect on human MRN, nor do the models available offer a clear insight into how the MIN/iDDR motif might affect ATP hydrolysis by RAD50. The ATP-bound form of RAD50 is proficient for DNA binding (70,71) (72), with DNA binding promoting ATM/Tel1 kinase activation (17,73), besides being a pre-requisite for tethering (22). Interestingly, the MIN motif of RIf2 has recently been proposed to disrupt Tel1 association with MRX and DSBs, not MRX association with DSBs (54). Our novel observation that the iDDR is capable of interfering with RAD50 DNA binding activity offers a rationale for interpreting the pleiotropic effects of the Rif2 MIN in yeast cells and opens the possibilities that these convergent MRN-disabling telomeric motifs might have multiple mechanisms of action in common (53). Further work will be required to fully elucidate the precise role of the iDDR and MIN motifs in the modulation of MRN activity at telomeres, but the analyses that we present here underscore how this is a ‘problem’ that has been addressed multiple times at telomeres during evolution, with a similar solution.

## Supplementary Material

gkae509_Supplemental_File

## Data Availability

The top-ranked AlphaFold2 models have been deposited in FigShare and can be accessed at https://www.doi.org/10.25377/sussex.22491634. All other data generated in this study are available from corresponding authors on reasonable request.
