# Supplementary material for: Binding of the TRF2 iDDR motif to RAD50 highlights a convergent evolutionary strategy to inactivate MRN at telomeres
Source: Nucleic Acids Res. 2024 Jun 17;52(13):7704–19. doi: 10.1093/nar/gkae509 (PMC11260466; doi:10.1093/nar/gkae509)
Supplement: gkae509_Supplemental_File [file gkae509_supplemental_file.pdf]

Binding of the TRF2 iDDR motif to RAD50 highlights a convergent evolutionary strategy to inactivate MRN at telomeres

Freddy Khayat[1,†], Majedh Alshmery[1,2,†], Mohinder Pal[1,3], Antony W. Oliver[1,*] and Alessandro Bianchi[1,*]
[1] Genome Damage and Stability Centre, School of Life Sciences, University of Sussex, Brighton, UK
[2] Department of LifeSciences, Hafr Al Batin University, SaudiArabia
[3] School of Biosciences, University of Kent, Canterbury, UK
To whom correspondence should be addressed. Tel: +441273872673; Fax:+441273678121; Email: a.bianchi@sussex.ac.uk
Correspondence may also be addressed to AntonyW.Oliver. Tel: +441273678349; Fax:+441273678121; Email: Antony.Oliver@sussex.ac.uk
[†] The first two authors should be regarded as Joint First Authors.

FIGURE LEGENDS

Supplemental Figure S1. Analysis of binding of RAD50 variants to CtIP.

Yeast two-hybrid analysis of binding of a CtIP fragment (845-890) to a panel of RAD50 mutants, as indicated, under variable stringency (on plates lacking histidine, in the absence or presence of 4mM 3AT).

Supplemental Figure S2. Analysis of binding of RAD50 variants to CtIP-F856A.

Yeast two-hybrid analysis of binding of a CtIP fragment (845-890) with a Phe856Ala mutation to a panel of RAD50 mutants, as indicated, under variable stringency (on plates lacking histidine, in the absence or presence of 4mM 3AT).

Supplemental Figure S3. Analysis of binding of RAD50 variants to CtIP-R877E.

Yeast two-hybrid analysis of binding of a CtIP fragment (845-890) with a Arg856Glu mutation to a panel of RAD50 mutants, as indicated, under variable stringency (on plates lacking histidine, in the absence or presence of 4mM 3AT).

Supplemental Figure S4. Analysis of binding of RAD50 variants to CtIP-T847K.

Yeast two-hybrid analysis of binding of a CtIP fragment (845-890) with a Thr856Lys mutation to a panel of RAD50 mutants, as indicated, under variable stringency (on plates lacking histidine, in the absence or presence of 4mM 3AT).

Supplemental Figure S5. Analysis of binding of RAD50 variants to CtIP-T859K.

Yeast two-hybrid analysis of binding of a CtIP fragment (845-890) with a Thr859Lys mutation to a panel of RAD50 mutants, as indicated, under variable stringency (on plates lacking histidine, in the absence or presence of 4mM 3AT).

Supplemental Figure S6. The iDDR perturbs DNA-binding by RAD50.

(**A**) SDS-PAGE electrophoresis of purified proteins, as indicated, used in the experiments in Figures 7A, 7B, S6B.

(B) (Top) Addition of iDDR(WT) perturbs the ability of HsRAD50 to bind DNA. Mutation of Trp465 to alanine (W465A) abrogates this effect. (Bottom) Quantitation of replicate EMSA experiments (three individual experiments). Error bars represent one standard deviation.

Table S1. Analysis of RAD50/CtIP interaction in double mutants.

	LTH					LTH + 4 mm 3AT				
	CtIP variant					CtIP variant				
**RAD50 mutation**	WT	F856A	R877E	T847K	T859K	WT	F856A	R877E	T847K	T859K
WT	+++	+++	+++	+++	+++	+++	+	+	-	-
empty	-	-	-	-	-	-	-	-	-	-
R3A	+++	+++	+++	+++	+++	+++	+	+	-	+
E5A	+++	+++	+++	-	-	+	-	+	-	-
K6A	+++	+++	+++	+++	+++	+++	+	+	+	+
S8A	+++	+++	+++	+++	+++	+++	-	+	-	-
L10A	+++	+++	+++	+++	+++	+++	+	+	-	+
S14A	+++	+++	+++	+++	+++	+++	+	+	-	+
I17A	+++	+++	+++	+++	+++	+++	-	+	-	-
E18A	+++	+++	+++	+++	+++	+++	+	+	-	-
D19A	+++	+++	+++	+++	+++	+	-	+	-	-
K22A	+++	+++	+++	+++	+++	+++++	+++++	+++	+	+++++
K22E	+++	+++	+++	+++	+++	+++++	+++++	+++	+++	+++++
Q23A	+++	+++	+++	+++	+++	+++	+	+	-	-
I24A	+++	+++	+++	+++	+++	+	-	+	-	-
T26A	+++	+++	+++	+++	+++	+++	+	+	-	+
E75A	+++	+++	+++	+++	+++	+++	+++	+++	-	+
D77A	+++	+++	+++	+++	+++	+++++	+++++	+++++	+++++	+++
R79A	+++	+++	+++	+++	+++	+++	+	+	+	+
R83A	+++	+++	+++	+++	+++	+++	+	+	-	+
R83E	+++	+++	+++	+++	+++	-	-	-	+	-
Q85A	+++	+	+++	-	-	-	-	-	-	-
L93A	+++	+++	+++	+++	+++	+	-	+	-	-
A95Q	+++	+++	+++	+++	+++	+++	+	+	-	-
Q97A	+++	+++	+++	+++	+++	+	-	+	-	-
S99A	+++	+++	+++	+++	+++	+++	+	+	-	-
V101A	+++	+++	+++	+++	+++	+++	+++	+	-	+
K112A	+++	+	+	-	+	-	-	-	-	-
E115A	+++	+++	+++	+++	+++	+++++	+++++	+++++	+++++	+++++
V117A	+++	+++	+++	+++	+++	+	-	-	-	-
T119A	+++	+++	+++	+++	+++	+	-	-	-	-
K126A	+++	+++	+++	+++	+++	+++	-	-	-	+
S128A	+++	+++	+++	+++	+++	+++	-	-	-	+
E1298K	+++	+++	+++	+++	+++	+++	+	-	-	-
E1298R	+++	+++	+++	+++	+++	+++	+	-	-	+

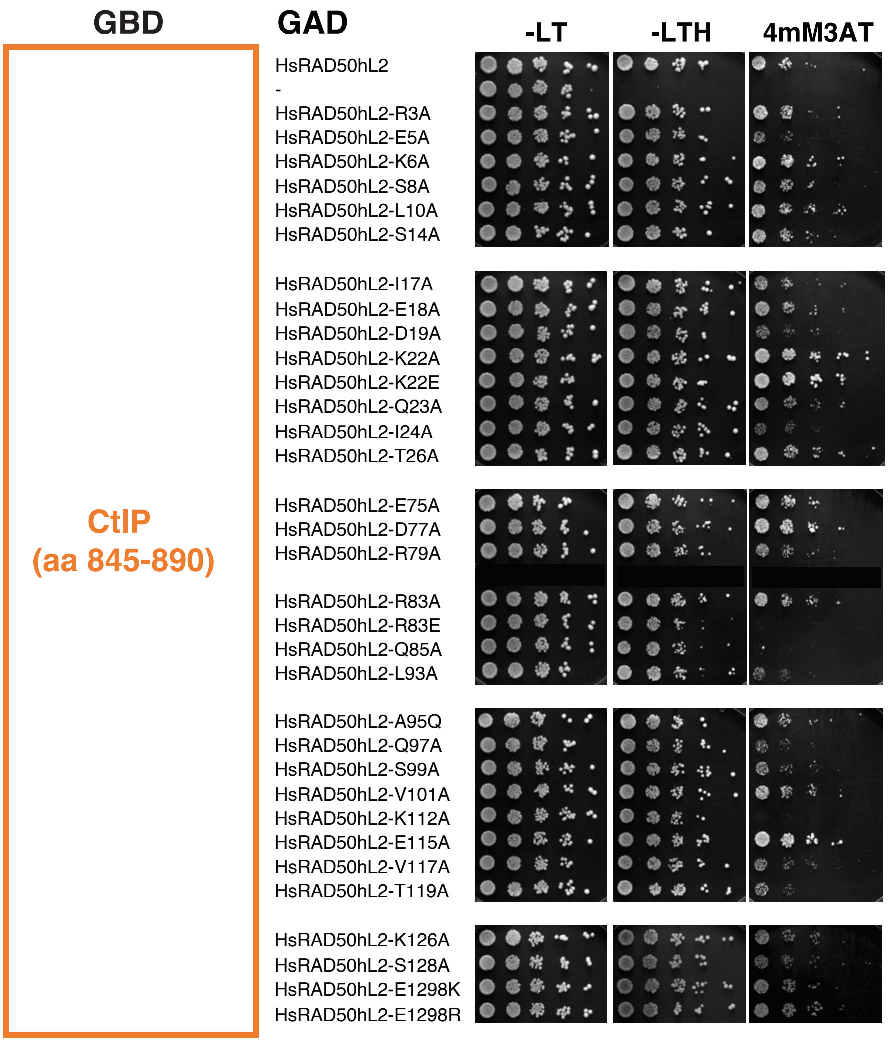
GBD
GAD
-LT
-LTH
4mM3AT
CtIP
(aa 845-890)
HsRAD50hL2
-
HsRAD50hL2-R3A
HsRAD50hL2-E5A
HsRAD50hL2-K6A
HsRAD50hL2-S8A
HsRAD50hL2-L10A
HsRAD50hL2-S14A
HsRAD50hL2-I17A
HsRAD50hL2-E18A
HsRAD50hL2-D19A
HsRAD50hL2-K22A
HsRAD50hL2-K22E
HsRAD50hL2-Q23A
HsRAD50hL2-I24A
HsRAD50hL2-T26A
HsRAD50hL2-E75A
HsRAD50hL2-D77A
HsRAD50hL2-R79A
HsRAD50hL2-R83A
HsRAD50hL2-R83E
HsRAD50hL2-Q85A
HsRAD50hL2-L93A
HsRAD50hL2-A95Q
HsRAD50hL2-Q97A
HsRAD50hL2-S99A
HsRAD50hL2-V101A
HsRAD50hL2-K112A
HsRAD50hL2-E115A
HsRAD50hL2-V117A
HsRAD50hL2-T119A
HsRAD50hL2-K126A
HsRAD50hL2-S128A
HsRAD50hL2-E1298K
HsRAD50hL2-E1298R

Figure S1

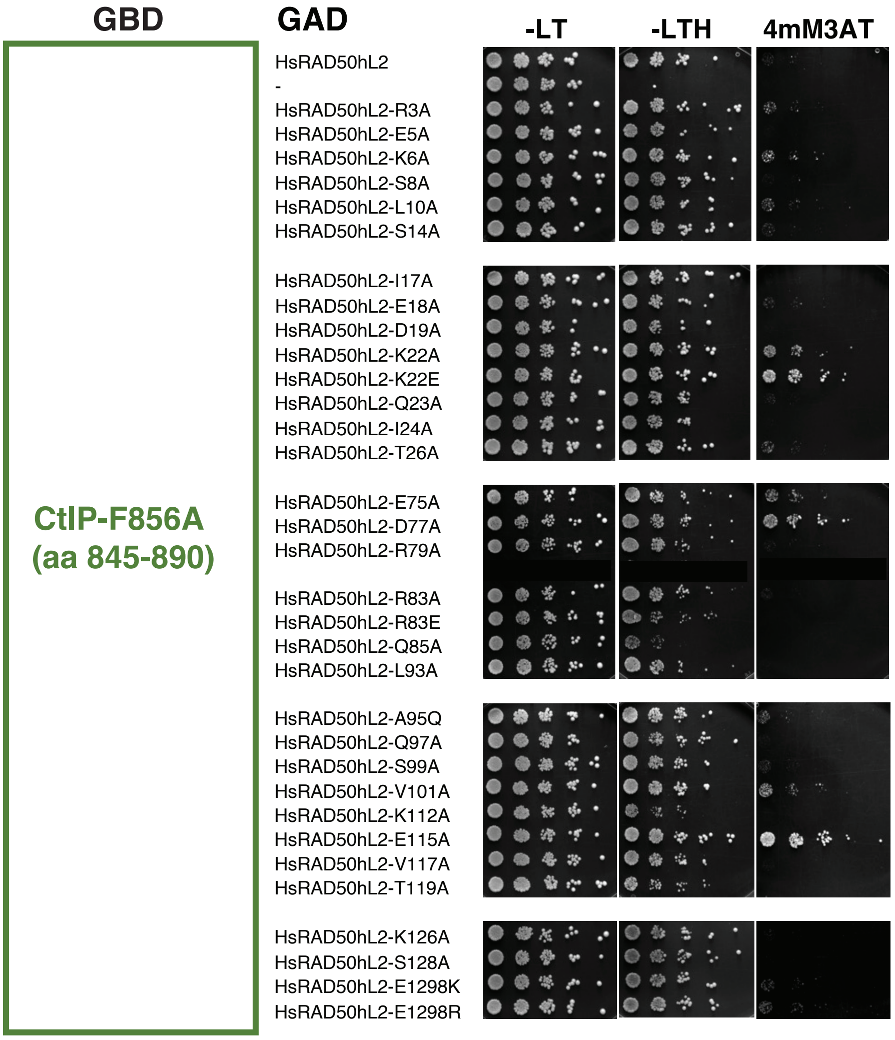
GBD
GAD
-LT
-LTH
4mM3AT
CtIP-F856A
(aa 845-890)
HsRAD50hL2
-
HsRAD50hL2-R3A
HsRAD50hL2-E5A
HsRAD50hL2-K6A
HsRAD50hL2-S8A
HsRAD50hL2-L10A
HsRAD50hL2-S14A
HsRAD50hL2-I17A
HsRAD50hL2-E18A
HsRAD50hL2-D19A
HsRAD50hL2-K22A
HsRAD50hL2-K22E
HsRAD50hL2-Q23A
HsRAD50hL2-I24A
HsRAD50hL2-T26A
HsRAD50hL2-E75A
HsRAD50hL2-D77A
HsRAD50hL2-R79A
HsRAD50hL2-R83A
HsRAD50hL2-R83E
HsRAD50hL2-Q85A
HsRAD50hL2-L93A
HsRAD50hL2-A95Q
HsRAD50hL2-Q97A
HsRAD50hL2-S99A
HsRAD50hL2-V101A
HsRAD50hL2-K112A
HsRAD50hL2-E115A
HsRAD50hL2-V117A
HsRAD50hL2-T119A
HsRAD50hL2-K126A
HsRAD50hL2-S128A
HsRAD50hL2-E1298K
HsRAD50hL2-E1298R

Figure S2

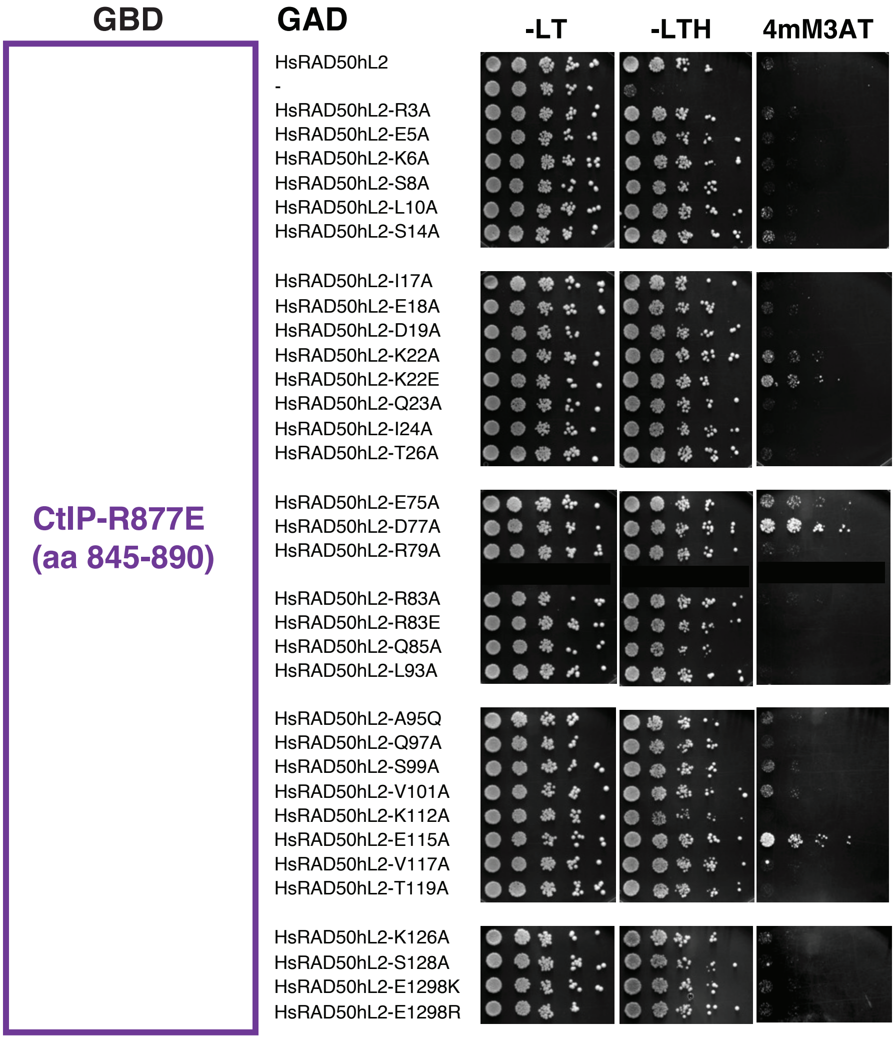
GBD
GAD
-LT
-LTH
4mM3AT
CtIP-R877E
(aa 845-890)
HsRAD50hL2
-
HsRAD50hL2-R3A
HsRAD50hL2-E5A
HsRAD50hL2-K6A
HsRAD50hL2-S8A
HsRAD50hL2-L10A
HsRAD50hL2-S14A
HsRAD50hL2-I17A
HsRAD50hL2-E18A
HsRAD50hL2-D19A
HsRAD50hL2-K22A
HsRAD50hL2-K22E
HsRAD50hL2-Q23A
HsRAD50hL2-I24A
HsRAD50hL2-T26A
HsRAD50hL2-E75A
HsRAD50hL2-D77A
HsRAD50hL2-R79A
HsRAD50hL2-R83A
HsRAD50hL2-R83E
HsRAD50hL2-Q85A
HsRAD50hL2-L93A
HsRAD50hL2-A95Q
HsRAD50hL2-Q97A
HsRAD50hL2-S99A
HsRAD50hL2-V101A
HsRAD50hL2-K112A
HsRAD50hL2-E115A
HsRAD50hL2-V117A
HsRAD50hL2-T119A
HsRAD50hL2-K126A
HsRAD50hL2-S128A
HsRAD50hL2-E1298K
HsRAD50hL2-E1298R

Figure S3

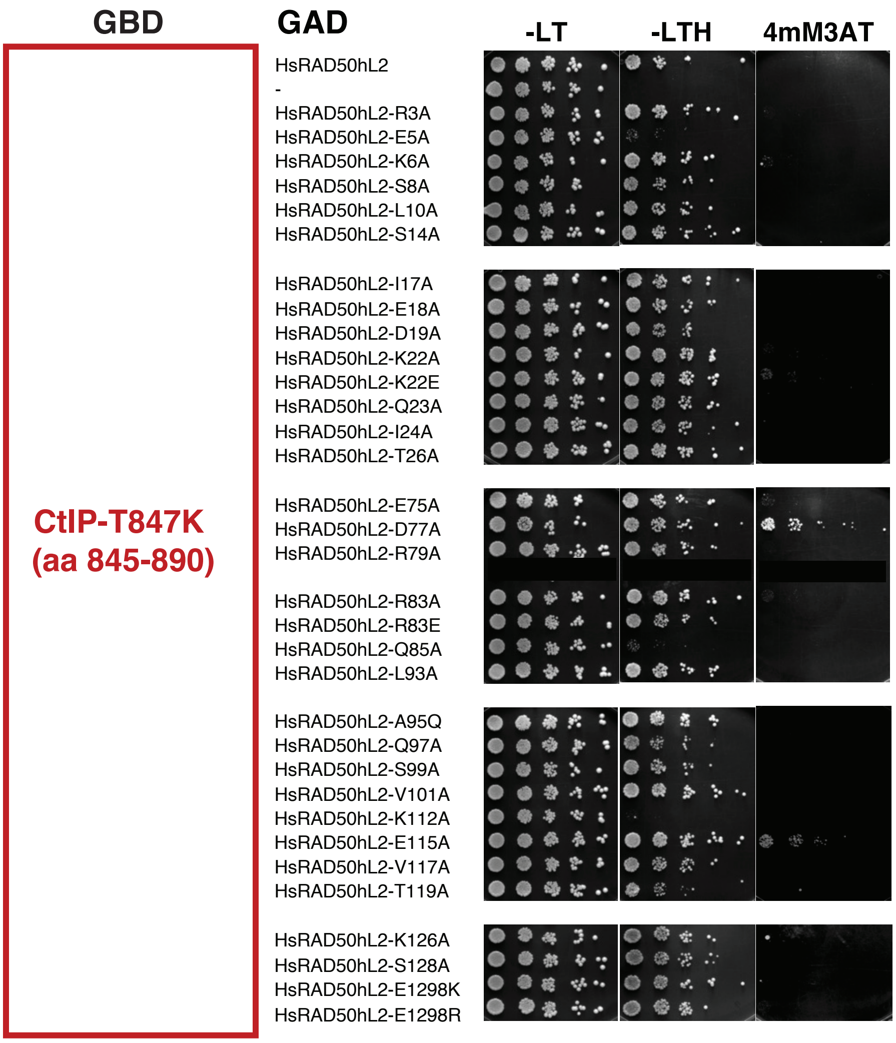
GBD
GAD
-LT
-LTH
4mM3AT
CtIP-T847K
(aa 845-890)
HsRAD50hL2
-
HsRAD50hL2-R3A
HsRAD50hL2-E5A
HsRAD50hL2-K6A
HsRAD50hL2-S8A
HsRAD50hL2-L10A
HsRAD50hL2-S14A
HsRAD50hL2-I17A
HsRAD50hL2-E18A
HsRAD50hL2-D19A
HsRAD50hL2-K22A
HsRAD50hL2-K22E
HsRAD50hL2-Q23A
HsRAD50hL2-I24A
HsRAD50hL2-T26A
HsRAD50hL2-E75A
HsRAD50hL2-D77A
HsRAD50hL2-R79A
HsRAD50hL2-R83A
HsRAD50hL2-R83E
HsRAD50hL2-Q85A
HsRAD50hL2-L93A
HsRAD50hL2-A95Q
HsRAD50hL2-Q97A
HsRAD50hL2-S99A
HsRAD50hL2-V101A
HsRAD50hL2-K112A
HsRAD50hL2-E115A
HsRAD50hL2-V117A
HsRAD50hL2-T119A
HsRAD50hL2-K126A
HsRAD50hL2-S128A
HsRAD50hL2-E1298K
HsRAD50hL2-E1298R

Figure S4

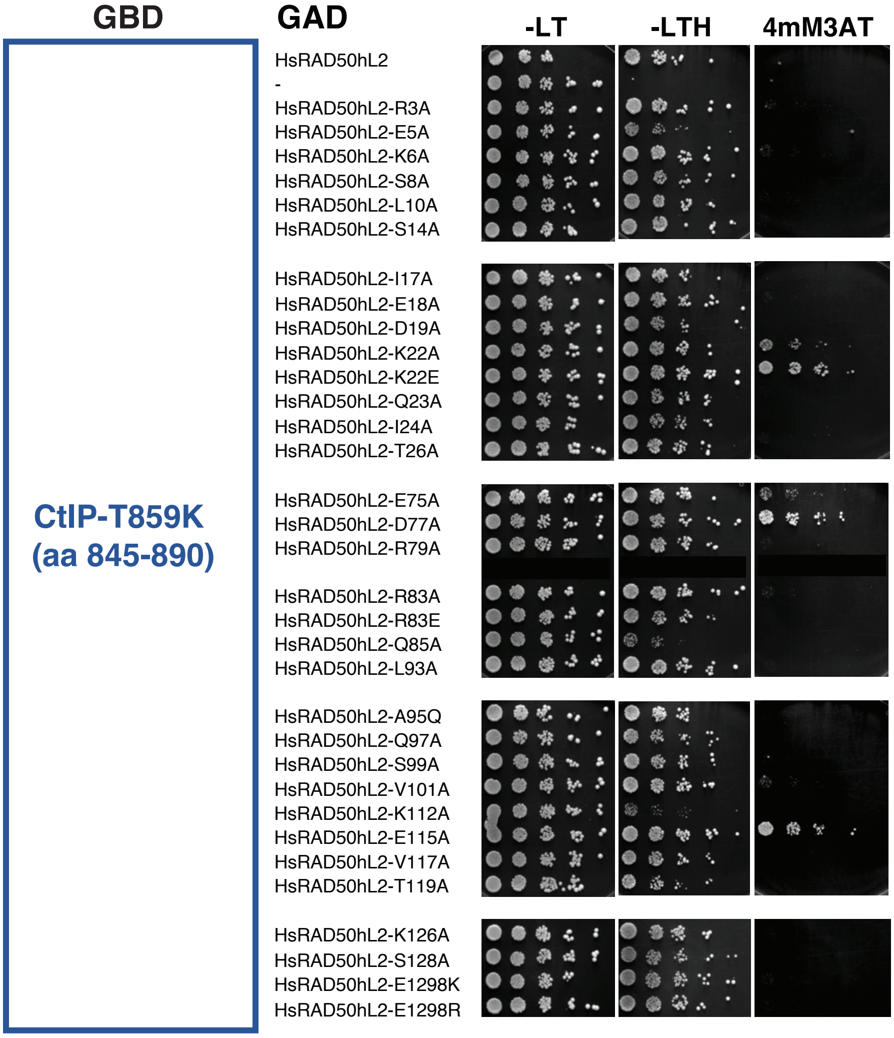
GBD
GAD
-LT
-LTH
4mM3AT
CtIP-T859K
(aa 845-890)
HsRAD50hL2
-
HsRAD50hL2-R3A
HsRAD50hL2-E5A
HsRAD50hL2-K6A
HsRAD50hL2-S8A
HsRAD50hL2-L10A
HsRAD50hL2-S14A
HsRAD50hL2-I17A
HsRAD50hL2-E18A
HsRAD50hL2-D19A
HsRAD50hL2-K22A
HsRAD50hL2-K22E
HsRAD50hL2-Q23A
HsRAD50hL2-I24A
HsRAD50hL2-T26A
HsRAD50hL2-E75A
HsRAD50hL2-D77A
HsRAD50hL2-R79A
HsRAD50hL2-R83A
HsRAD50hL2-R83E
HsRAD50hL2-Q85A
HsRAD50hL2-L93A
HsRAD50hL2-A95Q
HsRAD50hL2-Q97A
HsRAD50hL2-S99A
HsRAD50hL2-V101A
HsRAD50hL2-K112A
HsRAD50hL2-E115A
HsRAD50hL2-V117A
HsRAD50hL2-T119A
HsRAD50hL2-K126A
HsRAD50hL2-S128A
HsRAD50hL2-E1298K
HsRAD50hL2-E1298R

Figure S5

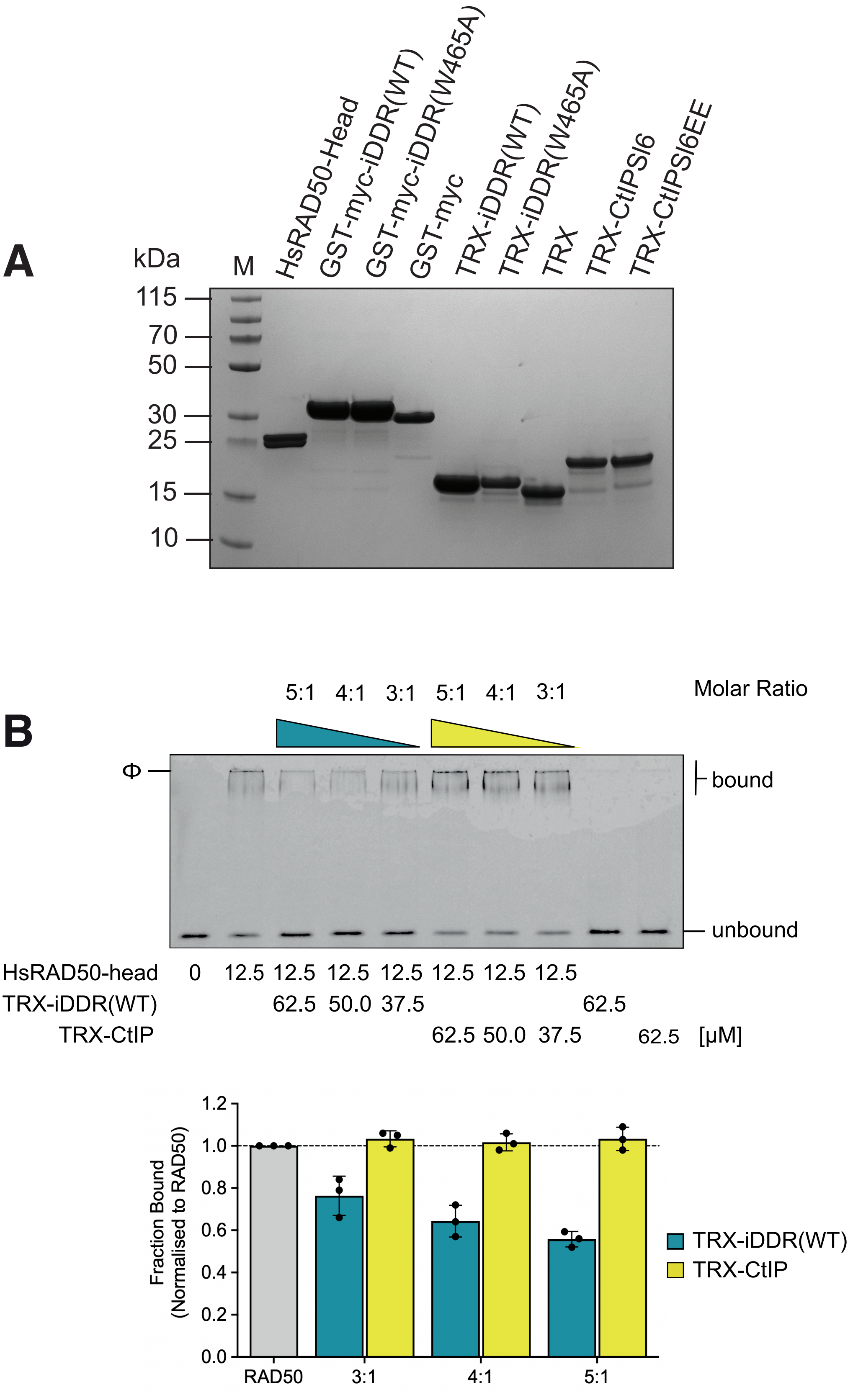
A
kDa
M
HsRAD50-Head
GST-myc-iDDR(WT)
GST-myc-iDDR(W465A)
GST-myc
TRX-iDDR(WT)
TRX-iDDR(W465A)
TRX
TRX-CtIPSI6
TRX-CtIPSI6EE
115
70
50
30
25
15
10
B
Molar Ratio
5:1 4:1 3:1 5:1 4:1 3:1
Φ
bound
unbound
HsRAD50-head 0 12.5 12.5 12.5 12.5 12.5 12.5 12.5
TRX-iDDR(WT) 62.5 50.0 37.5 62.5
TRX-CtIP 62.5 50.0 37.5 62.5 [μM]
Fraction Bound
(Normalised to RAD50)
1.2
1.0
0.8
0.6
0.4
0.2
0.0
RAD50
3:1
4:1
5:1
TRX-iDDR(WT)
TRX-CtIP

Figure S6
